# Neuroanatomy of the late Cretaceous *Thescelosaurus neglectus* (Neornithischia: Thescelosauridae) reveals novel ecological specialisations within Dinosauria

**DOI:** 10.1038/s41598-023-45658-3

**Published:** 2023-11-06

**Authors:** David J. Button, Lindsay E. Zanno

**Affiliations:** 1 Bristol Palaeobiology Group, School of Earth Sciences, University of Bristol, Bristol, BS8 1TQ UK; 2 Paleontology, North Carolina Museum of Natural Sciences, Raleigh, NC USA; 3 Department of Biological Sciences, North Carolina State University, Raleigh, NC USA

**Keywords:** Palaeontology, Evolution

## Abstract

Ornithischian dinosaurs exhibited a diversity of ecologies, locomotory modes, and social structures, making them an ideal clade in which to study the evolution of neuroanatomy and behaviour. Here, we present a 3D digital reconstruction of the endocranial spaces of the latest Cretaceous neornithischian *Thescelosaurus neglectus*, in order to interpret the neuroanatomy and paleobiology of one of the last surviving non-avian dinosaurs. Results demonstrate that the brain of *Thescelosaurus* was relatively small compared to most other neornithischians, instead suggesting cognitive capabilities within the range of extant reptiles. Other traits include a narrow hearing range, with limited ability to distinguish high frequencies, paired with unusually well-developed olfactory lobes and anterior semicircular canals, indicating acute olfaction and vestibular sensitivity. This character combination, in conjunction with features of the postcranial anatomy, is consistent with specializations for burrowing behaviours in the clade, as evidenced by trace and skeletal fossil evidence in earlier-diverging thescelosaurids, although whether they reflect ecological adaptations or phylogenetic inheritance in *T. neglectus* itself is unclear. Nonetheless, our results provide the first evidence of neurological specializations to burrowing identified within Ornithischia, and non-avian dinosaurs more generally, expanding the range of ecological adaptations recognized within this major clade.

## Introduction

Reconstructing the ecology and behaviour of fossil taxa relies upon multiple lines of evidence and inference^1^ including paleoneurology, the study of the brain and other nervous tissues of extinct animals^2,3^. The neurology of extinct taxa can be investigated through study of endocasts taken from the internal surfaces of the cranial vault^2,3^, representing the surface of the dural envelope, providing information on the size and structure of the brain and its major regions, variables intrinsically linked to sensory perception, cognition, and behaviour^2–6^. Similarly, the shape of the endosseous labyrinth of the inner ear informs reconstruction of equilibrium perception^7,8^, locomotory behavior^7^, and hearing range^9^. Together, these data provide valuable information on organismal paleobiology and evolutionary patterns in sensorineural anatomy accompanying ecological and behavioural transitions observed in the fossil record (e.g.^2,10–13^).

Ornithischian dinosaurs expressed remarkable diversity in body size^14^, trophic adaptations^15^, climatic range^16^, gait^17^, and social interactions (^18,19^, and references therein), and trace and body fossils demonstrate specific behaviours such as flocking (e.g.^20,21^) and burrowing^22^. Consequently, Ornithischia is an ideal clade in which to investigate sensorineural patterns associated with behavioural evolution^23–25^. However, whereas endocasts are relatively well-known from thyreophorans, ceratopsians, and iguanodontian ornithopods (^26^ and references therein), they are more sparsely sampled across the remainder of Ornithischia.

Here, we present a three-dimensional endocranial reconstruction based upon CT-scanning of the skull of NCSM 15728 (‘Willo’), a specimen of the latest-Cretaceous^27^ neornithischian *Thescelosaurus neglectus* (Gilmore^28^). CT-scanning and virtual segmentation carry many advantages over classical techniques in palaeoneurology, allowing extraction of fine-scale information^29^ and virtual restoration of deformed braincases^30^. Although known for over a century from multiple specimens encompassing most of the skeleton^31^, the biology and ecology of *Thesceosaurus* remain enigmatic. It is unusual in both its large size^27^ and robust proportions^32,33^ relative to phylogenetically proximate taxa, and assessments of its locomotory behaviour have ranged from an agile and cursorial^34^ through graviportal^33,35–37^ biped, or even as facultatively quadrupedal^33^. The phylogenetic position of *Thescelosaurus* is similarly controversial^38^, considered either a late-surviving non-iguanodontian ornithopod (e.g.^38–41^), or as the eponymous member of a relatively poorly-understood family of non-cerapodan neornithischians, the Thescelosauridae (e.g.^42–46^). To date, no digital endocasts have been generated for any putative thescelosaurids, whereas physical endocasts are either incomplete and provide limited information^47,48^ or are known^48^ from a probable juvenile^27^ (the holotype of *T. assiniboiensis*^27^). The latter is problematic, as ornithischian endocasts are known to vary considerably through ontogeny^49^. Consequently, our results help to illuminate endocranial anatomy in an under-sampled region of the ornithischian tree; elucidate the biology of one of the last-surviving, but poorly-understood, non-avian dinosaurs; and inform the ecological range present among dinosaur taxa immediately prior to the end-Cretaceous mass extinction.

## Institutional abbreviations

AMNH—American Museum of Natural History, New York, USA. CMN—Canadian Museum of Nature, Ottawa, Canada. MNHN—Muséum national d’Histoire Naturelle, Paris, France. NCSM—North Carolina Museum of Natural Sciences, Raleigh, USA. PKUP—Peking University Palaeontological Collections, Beijing, China. RBINS—Royal Belgian Institute of Natural Sciences, Brussels, Belgium. ROM—Royal Ontario Museum, Toronto, Canada. YPM—Yale Peabody Museum, New Haven, USA.

## Results

### Endocast reconstruction

**Figure 1:**
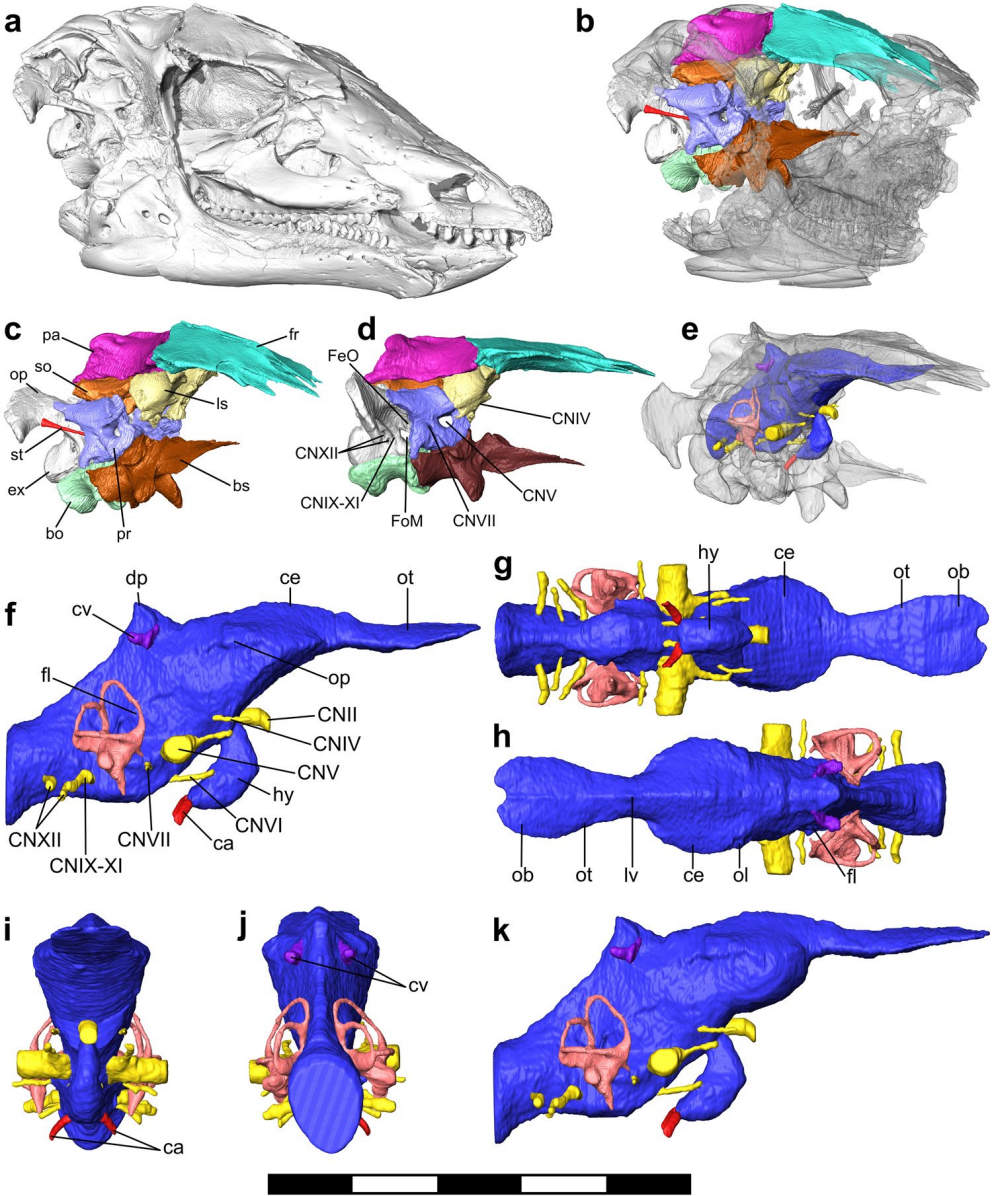
Reconstructed skull, braincase, and endocast of NCSM 15728. (**a**) Surface render of the skull of NCSM 15728 in oblique right lateral view. (**b**) Posterior half of the skull in oblique right lateral view, with the segmented bones of the braincase and skull roof in color and the other skull elements as translucent. (**c**) Segmented braincase as preserved in oblique right lateral view. d) Retrodeformed braincase in right lateral view. (**e**) Reconstructed endocast within the braincase, with the dural envelope in blue, endosseous labyrinth in pink, cranial nerves in yellow, arteries in red, veins in purple, and surrounding bones as translucent. (**f**–**j**) Endocast with minimum estimated size of the cerebrum, in right lateral (**f**), ventral (**g**), dorsal (**h**), anterior (**i**), and posterior (**j**) views. (**k**) Endocast with maximum estimated cerebrum in right lateral view. Abbreviations as follows: bo = basioccipital, bs = fused basisphenoid and parasphenoid rostrum, ca = carotid artery, ce = cerebral hemispheres, CN = cranial nerve/cranial nerve exit, cv = caudal middle cerebral vein, dp = dural peak, ex = exoccipital, FeO = fenestra ovalis, FeM = foramen metoticum, fl = flocculus, fr = frontal, hy = hypophysis, ls = laterosphenoid, lv = longitudinal venous sinus, ob = olfactory bulb, op = opisthotic, ol = optic lobe, ot = olfactory tract, pa = parietal, pr = prootic, so = supraoccipital, st = stapes. CN = cranial nerve, ca = carotid artery, cv = caudal middle cerebral vein. Scale bar = 200 mm for a-e and 100 mm for (**f**–**k**).

**Figure 2:**
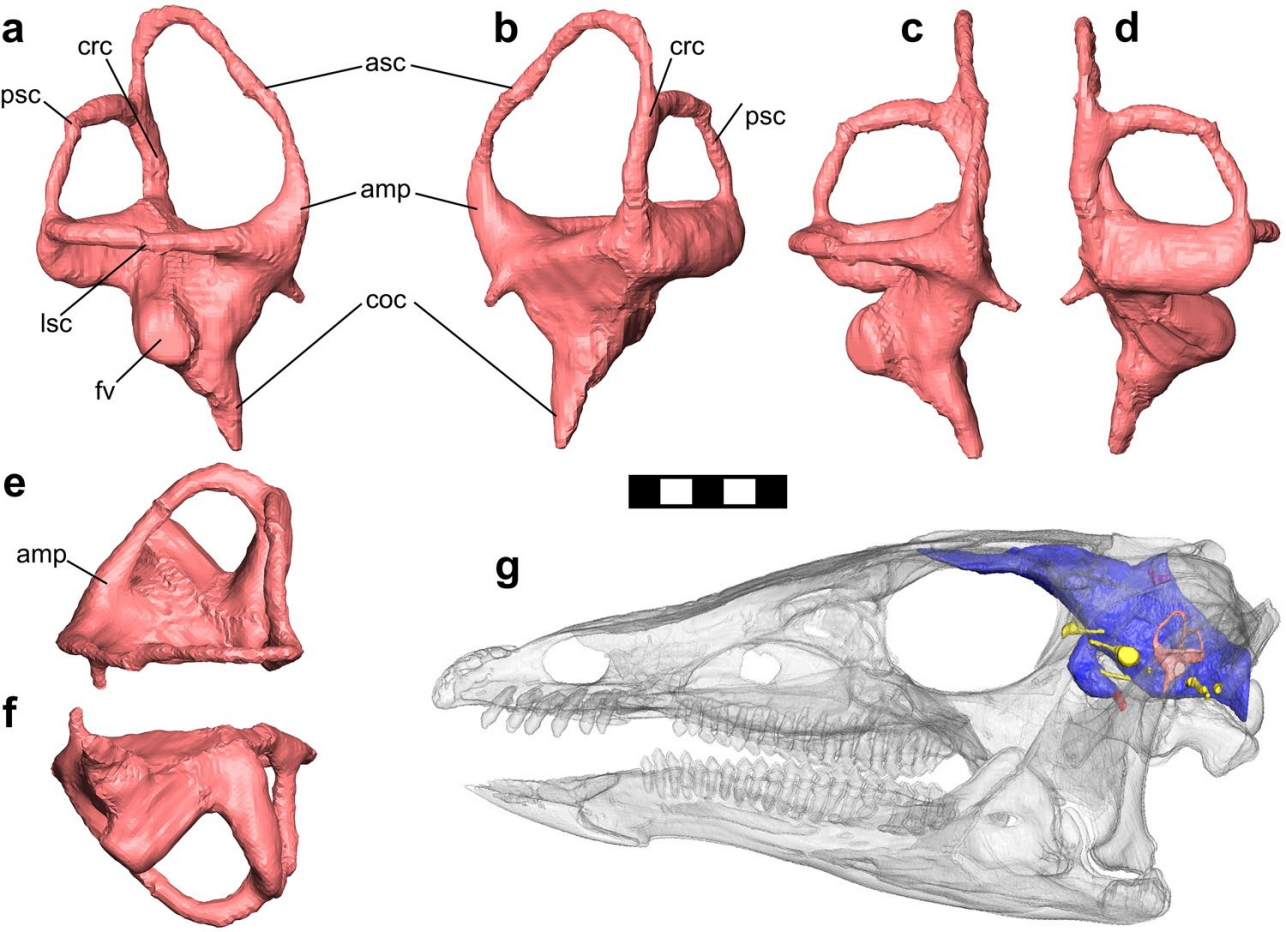
Reconstructed endosseous labyrinth of NCSM 15728. (**a**–**f**) Right labyrinth in lateral (**a**), medial (**b**), dorsal (**c**), ventral (**d**), anterior (**e**), and posterior (**f**) views. (**g**) Restored skull oriented in the “alert posture”. Abbreviations as follows: amp = ampulla, asc = anterior semicircular canal, coc = endosseous cochlear duct (lagena), crc = crus communis, fv = vestibule of inner ear, lsc = lateral semicircular canal, psc = posterior semicircular canal. Scale bar = 10 mm for a-f and 50 mm for g.

The skull of NCSM 15728 (Fig. 1a) has suffered some ventrolateral shearing, leading to partial disarticulation of the braincase (Fig. 1a–c). We therefore retrodeformed^30^ the braincase to accurately portray its original dimensions (Fig. 1d, e) and, by extension, the original shape of the endocranial spaces (see "Methods"). This permits reconstruction of a cranial endocast, representing the surface of the dural envelope (Fig. 1e–k) and the endosseous labyrinth of the inner ear (Fig. 2). Representative measurements of the endocast are given in Supplementary Table S1, and detailed description and comparisons of endocranial morphology are provided in the supplementary information and supplementary figures S1–S3.

### Relative brain size

The Encephalization Quotient (EQ) provides a measure of the overall brain size of an organism relative to its mass^5,6^. The calculated reptile encephalization quotient^50^ (REQ) range for *T. neglectus* indicates its brain was of average or below-average size for a reptile of its mass, smaller than those reported from all other neornithischians other than ceratopsids, and most similar to those of *Triceratops* and thyreophorans (Table 1). Even assuming a greater 60%^51^ or 73%^25^ endocranial fill, the REQ of *T. neglectus* still falls within the range of extant reptiles and below those observed in non-ceratopsid ceratopsians and ornithopods, as well as that estimated for *Leaellynasaura* (1.1–1.8^52^), although the probable juvenile status of the latter specimen limits the paleoneurological conclusions that can be drawn from it^53^. To ensure comparability of results, REQs were re-calculated for other ornithischian taxa using updated brain tissue density, endocranial fill, and body mass estimates, where necessary (see "Methods"). Re-calculated REQs of these taxa remain broadly similar to previous estimates, although with slight differences due to differences in the density of brain tissues and body masses used herein (Table 1).

**Table 1:** Reptile encephalization quotient (REQ) values calculated for a range of ornithischian taxa.

Taxon	Body mass/kg	Endocast volume /ml	REQ		Source
			50% fill	60% fill	
*Thescelosaurus neglectus*	3.39E + 02^a^	27.25–28.61	0.797–0.840	0.956–1.00	This study
*Kentrosaurus aethiopicus*	1.60E + 03^a^	48	0.595	0.715	^6,50,69^
*Stegosaurus stenops*	2.00E + 03^b^-6.95E + 03^a^	45–56	0.387–0.481	0.464–0.577	^4,50,69^
*Euoplocephalus tutus*	2.33E + 03^a^	82	0.825	0.991	^6,50,69^
*Camptosaurus dispar*	4.00E + 02^b^	46	1.23	1.472	^4,50^
*Lurdusaurus arenatus*	4.19E + 03^a^	167	1.215	1.458	^96^
*Proa valdearinnoensis*	3.56E + 03	316	2.51	2.82	^25^
*Iguanodon bernissartensis*	8.27E + 03^a^	357	1.784	2.141	^96^
*Mantellisaurus atherfieldensis*	1.43E + 03^a^	131 +	1.73 +	2.07 +	^96^
*Edmontosaurus* sp.	3.40E + 03^b^-6.61E + 03^a^	300	1.70–2.45	2.04–2.94	^4,50,51^
*Amurosaurus riabinini*	4.79E + 03	290	1.96	2.35	^88^
*Hypacrosaurus altispinus*	3.69E + 03^a^	275.9	2.154	2.585	^23^
*Psittacosaurus lujiatunensis*	2.50E + 01	14.3	1.767	2.121	^158^
*Protoceratops andrewsi*	8.27E + 01^a^	30	1.913	2.296	^4,161^
*Triceratops* sp.	6.00E + 03^b^-13.54E + 03^a^	140	0.532–0.835	0.639–1.00	^4,50^

REQs were calculated for both 50% and 60% fills of the endocranial space by the brain.

^a^Body mass estimate derived from stylopodial circumferences by^14^.

^b^Body mass estimate derived from scale models by^163^. Other body masses were estimated from stylopodial circumferences by the authors listed in the source column.

### Olfactory tract size and olfactory ratio

**Figure 3:**
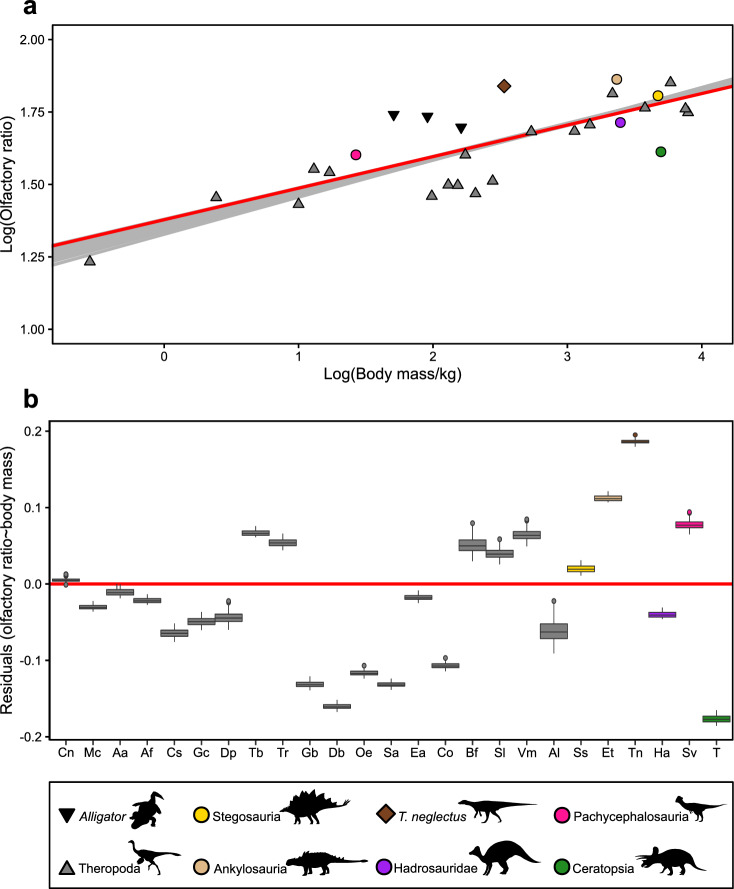
Comparison of olfactory ratio between *T. neglectus* and other archosaur taxa. a) Results of phylogenetic generalized least-squares regression of log_10_-transformed olfactory ratio against body mass, across 100 phylogenetic trees. The regression line from the best-performing iteration (model *p* = 2.34E−10, R^2^ = 0.831) is given in red, and the total range of regression lines across all topologies in grey (median *p* = 9.22E−09, R^2^ = 0.768). See Supplementary Information 3for full results. b) Boxplots of residuals from the 100 pgls regressions, with the medians given by midlines, whiskers equalling 1.5× the interquartile range, and outliers beyond this as points. Zero is marked by the horizontal red line. X-axis label abbreviations as follows: Cn = *Ceratosaurus nasicornis*, Mc = *Majungasaurus crenatissimus*, Aa = *Acrocanthosaurus atokensis*, Af = *Allosaurus fragilis*, Cs = *Carcharodontosaurus saharicus*, Gc = *Giganotosaurus carolinii*, Dp = *Dilong paradoxus*, Tb = *Tarbosaurus bataar*, Tr = *Tyrannosaurus rex*, Gb = *Garudimimus brevipes*, Db = “*Dromiceiomimus brevitertius*”, Oe = *Ornithomimus edmontonicus*, Sa = *Struthiomimus altus*, Ea = *Erlikosaurus andrewsi*, Co = *Citipati osmolskae*, Bf = *Bambiraptor feinbergi*, Sl = *Saurornitholestes langstoni*, Vm = *Velociraptor mongoliensis*, Al = *Archaeopteryx lithographica*, Ss = *Stegosaurus stenops*, Et = *Euoplocephalus tutus*, Tn = *Thescelosaurus neglectus*, Ha = *Hypacrosaurus altispinus*, Sv = *Stegoceras validum*, T = *Triceratops* sp. See Supplementary Table S2 for ornithischian olfactory ratio data.

The olfactory tract of *T. neglectus* is large, with the olfactory bulbs making up ~ 3% of the total endocast volume (Supplementary Table S1), exceeding the relative volume exhibited by extant birds (including *Apteryx*)^54^ and overlapping with values reported for rodents and lagomorphs^55^. The olfactory ratio provides a proxy for olfactory acuity in fossil taxa^56^. The calculated olfactory ratio of *T. neglectus* is also large, greater than observed in extant birds^57^ and more comparable in magnitude to those of *Euoplocephalus*, *Alligator*, and predatory theropods than to *Hypacrosaurus*, *Triceratops*, or herbivorous theropods (Fig. 3a, Supplementary Table S2). However, the olfactory tract exhibits a high degree of allometric independence from the rest of the brain^58^ making it difficult to generalise comparisons of absolute magnitudes across large phylogenetic scales. Phylogenetic generalised least squares (pgls) regressions of olfactory ratio against body mass were used to compare development of the olfactory tract among non-avian dinosaurs (see "Methods"). A significant relationship was retrieved regardless of the topology used (Fig. 3a): further, comparison of residuals indicates that *T. neglectus* did indeed have a substantially larger olfactory ratio than expected for its size, more so than any other sampled taxon (Fig. 3b).

### Hearing range

The calculated best hearing range^9^ of *T. neglectus* occupies a narrow low-frequency range of ~ 1854 Hz (approx. 296–2150 Hz), a frequency of best hearing^9,59^ of ~ 1100–1200 Hz, and an upper limit of hearing^59^ of 3051 Hz. This is robust to the choice of scaling relationship used, with best hearing frequency broadly similar whether derived from the length of the endosseous cochlear duct^9^ or estimated basilar papilla length^59^ (Supplementary Table S3). This hearing range is similar to those reported from some crocodilians (e.g., *Caiman crocodylus*, best hearing range = 300–2000 Hz, mean best hearing = 1150 Hz^60^) and squamates (e.g., *Chalcides occelatus*, best hearing range = 300–2000 Hz, mean best hearing = 1150 Hz^61^), but is lower than those of other small neornithischians (e.g. *Dysalotosaurus*, best hearing range =  ~ 350–3850 Hz, mean best hearing = 2100 Hz^62^, see Discussion and Supplementary Table S3), and extant birds^9^.

### Semicircular canals

**Figure 4:**
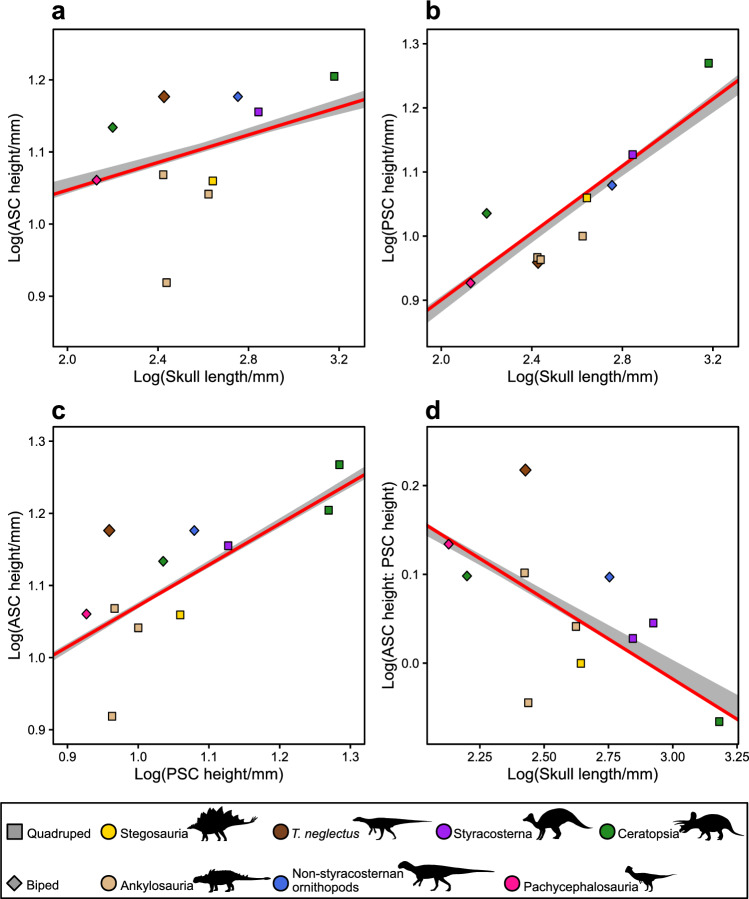
Results from pgls regressions of log_10_-transformed semicircular canal heights against skull length. (**a**) anterior semicircular canal (ASC) height against skull length (best-performing model *p* = 0.127, R^2^ = 0.266; median *p* = 0.193, R^2^ = 0.202). (**b**) posterior semicircular canal (PSC) height against skull length (best-performing model *p* = 0.000164, R^2^ = 0.846; median *p* = 0.000163, R^2^ = 0.846). (**c**) anterior semicircular canal height against posterior semicircular canal height (best-performing model *p* = 0.012, R^2^ = 0.481, median *p* = 0.0136, R^2^ = 0.472). (**d**) anterior canal height divided by posterior canal height, against skull length (best-performing model *p* = 0.00894, R^2^ = 0.551; median *p* = 0.0222, R^2^ = 0.458). Results are plotted by taxon and locomotor style (see Materials and methods for decisions on quadrupedal vs. bipedal taxa). Heights of the anterior and posterior semicircular canals measured as their maximum diameter measured perpendicular to the long axis of the lateral semicircular canal. All pgls regressions conducted across 100 phylogenetic trees: regression lines from the best performing of these iterations in red, the range across all trees given in grey.

*Thescelosaurus* exhibits a very long and slender anterior semicircular canal (ASC), relative to both the lateral (LSC) and posterior (PSC) semicircular canals. Comparison of semicircular canal height across Ornithischia reveals that *T. neglectus* has a tall ASC, low PSC, and large ASC height: PSC height ratio relative to its skull length (Fig. 4a–d), greater than that known from any other ornithischian (Fig. 4d). A significant relationship was resolved between PSC height and skull length but not ASC height and skull length (Fig. 4a, b).

Extant tetrapods generally orient the LSC horizontally when adopting a typical “alert” head posture (^29^ and references therein, but see^63^). Orienting the LSC horizontally in *T. neglectus* (Fig. 2g) results in a slightly upturned head posture, with the tip of the premaxilla lying flush with the foramen magnum, and the oral margin inclined at ~ 6°. This differs from the ventrally deflected alert postures reconstructed for ankylosaurs^64^, ceratopsians^65,66^, *Tenontosaurus*^67^, hadrosaurs (Figs. 2, 3, 4 in^23^) and many saurischians^29,68^, but similarly inclined postures have been reported for *Dysalotosaurus*^62^ and the sauropodomorph *Massospondylus*^68^.

## Discussion

### Sensory biology of *Thescelosaurus neglectus*

**Figure 5:**
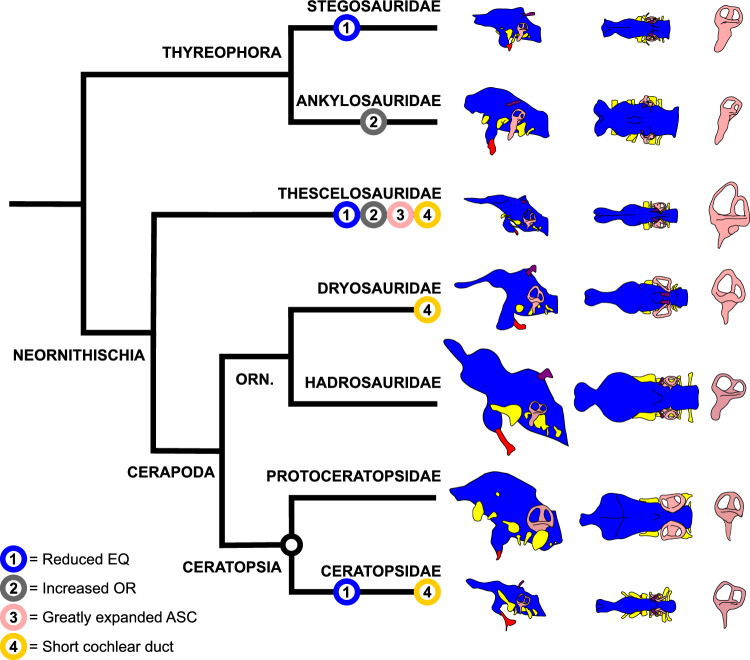
Comparison of the endocast of *T. neglectus* with other ornithischians. Simplified phylogeny of Ornithischia, after^42^. Endocasts (left) and endosseous labyrinths (right) are illustrated for (from top): the stegosaurid *Stegosaurus* (redrawn from^70^), the ankylosaurid *Euoplocephalus* (redrawn from^70^), the thescelosaurid *Thescelosaurus*, the dryosaurid *Dysalotosaurus* (redrawn from^49^), the hadrosaurid *Hypacrosaurus* (redrawn from^23^), the protoceratopsid *Protoceratops* (redrawn from^161^), and the ceratopsid *Pachyrhinosaurus* (redrawn from^172^). Endocast lengths are scaled in proportion to REQ^1/3^ for each taxon (Table 1), with the REQ of *Triceratops* used to approximate that of *Pachyrhinosaurus*. Endosseous labyrinth heights are scaled in proportion to the ASC height: PSC height ratio of each taxon. Distribution of sensorineural characters discussed in the text is indicated. Orn. = Ornithopoda.

The reconstructed endocast of *Thescelosaurus neglectus* exhibits a combination of characters that are plesiomorphic for Ornithischia (elongate olfactory tract, expanded cerebral hemispheres^69^), or at least widely distributed within the clade (short cochlear duct^62^, expansive dural peak^49,70^) (see Supplementary Information). The endocast of *T. neglectus* differs from those of other ornithischians primarily in characters related to its sensory biology and ecology, exhibiting a unique combination of a limited hearing range, large olfactory ratio, low REQ, and elongate ASC (Fig. 5).

The short cochlear duct of *T. neglectus* suggests limited ability to discriminate low and high-frequency sounds relative to many other ornithischian taxa. Its calculated best hearing range (~ 296–2150 Hz) is narrower than that reported for the dryosaurid *Dysalotosaurus* (~ 350–3850 Hz^62^), with *Thescelosaurus* exhibiting less sensitivity to higher frequencies, while also lacking the enhanced sensitivity toward low frequencies observed in lambeosaurines^23^. By contrast, the observed very high olfactory ratio, which correlates with olfactory acuity^56^, suggests an acute sense of smell in *Thescelosaurus*. Among ornithischians, comparably high olfactory ratios are also observed in ankylosaurs (Fig. 3a, b), for which manual surface-digging for buried food has been posited^71,72^. The robust forelimbs^33,73^ and rostrally fused premaxillae^74^ of *Thescelosaurus* could similarly have been used to unearth foodstuffs such as roots and tubers located via olfaction.

### Relative brain size and encephalisation quotient

Relative brain size and the encephalization quotient have long been considered to correlate positively with increased cognitive ability and behavioural complexity^4–6,50^, and empirical studies have linked greater relative size of the brain with increased performance in cognitive tasks such as learning^75,76^, memory^77^, problem-solving^78^, behavioural flexibility^79^, and innovation^80,81^. Increased absolute or relative brain size has also been widely linked to greater social cognition^80,82–84^, as required in larger^85^ (but see^86^), more complex^82,83^, or competitive^86,87^ groups, with the increased REQ and forebrain volume of styracosternan ornithopods likewise used to suggest large group sizes^25^ and complex social interaction^23,88^. Consequently, the ‘reptilian’ REQ of *T. neglectus* may indicate a cognitive and behavioural range within that of extant reptiles, and less complex social interactions and/or smaller group sizes than in other sampled Late Cretaceous ornithischians. This would be consistent with the short cochlear duct, implying a lack of vocalizations and, in-turn, small aggregation sizes^9^, in *Thescelosaurus*, and also its lack of bony ornaments for use in intraspecific signalling and combat, as present in many other ornithischian taxa (see^18,19^, and references therein). Multiple small, probable juvenile, individuals of *Thescelosaurus* are preserved in a multi-taxon bonebed from the ‘convenience store’ locality of the Frenchman Formation^27^, providing possible counter-evidence for larger aggregations. However, it is presently unknown if this association represents a genuine biological signal, or is instead the result of preservational biases^27^, and the total number of individuals is not reported. Among other thescelosaurids, multiple associations of 2–3 individuals, including adult-juvenile associations postulated to represent family groupings, are known from *Oryctodromeus*^22,89^ and a new taxon from the Mussentuchit Member of the Cedar Mountain Formation^90^. This lends some tenuous support to similarly small group size in *Thescelosaurus*, although it is possible these proximate small *Oryctodromeus* groups belonged to a larger colony^89^. Ultimately, hypotheses of group sizes in *Thescelosaurus* are difficult to test.

Furthermore, any estimation of the neuroanatomy and behaviour of fossil animals is difficult, and comparison of brain size measurements alone, without reference to neural circuitry, is an oversimplification^91,92^. Moreover, most comparative cognitive studies have focused on mammals, which may be problematic given the fundamental differences between the pallia of extant mammals and birds^91^. Indeed, complex behaviours and advanced cognitive skills are known from extant reptiles despite their relatively low EQs^93^, and the validity of EQ as a measure of ‘intelligence’ is doubtful^91,94,95^, with work on primates suggesting absolute brain size is instead a better predictor of cognitive performance^94,95^. Despite its smaller overall endocast size, comparison of brain regions indicates that the cerebral hemispheres—responsible for ‘higher’ cognitive functions^85^—occupy ~ 30% of the total endocast volume in *T. neglectus*, a greater proportion than in some iguanodontians such as *Dysalotosaurus* (~ 16%^25^, see Supplementary Table S4). This may be a consequence of the relatively smaller brain size of *Thescelosaurus*; more complex patterns of cerebrum evolution in Neornithischia than previously recognized; or, alternatively, independent expansion of the cerebral hemispheres—and so, by inference, cognitive capacity—in the lineage leading to *Thescelosaurus*, parallel with the stepwise increases in forebrain volume observed within Iguanodontia^23–25,88,96^. Nonetheless, the cerebral hemispheres of *T. neglectus* remain proportionately smaller than in *Proa* and most hadrosaurids (~ 40%^23–25^, see Supplementary Table S4). This, together with the absolutely smaller size of its endocast and lower REQ, suggests comparatively simple cognitive ability and less complex behaviours in *T. neglectus* than in coeval ornithopods, and the small absolute size of the endocast compared to ankylosaurids and neoceratopsians may also be notable.

### Endocranial anatomy and agility in *Thescelosaurus neglectus*

Since its discovery, the locomotory performance of *Thescelosaurus* has been controversial. Although originally reconstructed as an agile, cursorial animal on the basis of its bipedal skeletal proportions and size^34^, subsequent authors have typically considered *Thescelosaurus* to have been poorly adapted to running due to its overall robust build and the structure of the hindlimb^33,35,36^. Specifically, adult *Thescelosaurus* exhibit a longer femur than tibia, and relatively short metatarsals^32,33,35,37^, unlike extant cursorial mammals, cursorial theropods, and the cursorial neornithischians *Parksosaurus*, *Dryosaurus*, *Dysalotosaurus* and *Hypsilophodon*^33,37^. Instead, it exhibits proportions more comparable to those observed in large hadrosaurids^33^, and it has been suggested that *Thescelosaurus* represented an independent acquisition of graviportality^33,36,37^, or possibly even facultative quadrupedality^33^, parallel to that observed in iguanodontian ornithopods. Despite this, *Thescelosaurus* does differ from graviportal iguanodontians in other hindlimb characters such as the more proximal location of the fourth trochanter of the femur^33,37^, resulting in a lower moment arm for the caudofemoralis musculature and faster, but less efficient, retraction of the hindlimb, an adaptation towards fast running also seen in taxa such as *Parksosaurus*, *Hypsilophodon* and *Dryosaurus*^33,37^. However, the fourth trochanter of *Thescelosaurus* is still situated more distally than in other thescelosaurids such as *Koreanosaurus*^97^, indicating reduced relative hindlimb retraction speed, but greater power, relative to immediate outgroups. Consequently, the bulk of the evidence suggests reduced cursoriality and greater hindlimb retraction power in *Thescelosaurus* relative to earlier-diverging thescelosaurids and outgroups.

The dimensions of the flocculus may provide indirect evidence of agility as a proxy for the size of the floccular lobes, which are important in gaze stabilization through coordinating the vestibular system with the muscles of the eyes and neck^98,99^. The small, indistinct flocculus observed here (Fig. 1f) implies reduced agility in *Thescelosaurus*, especially when compared to the large flocculi of *Dryosaurus* and *Zephyrosaurus*^48^. However, flocculus size decreases through ontogeny in *Dysalotosaurus*^49^, and small flocculi are also observed in taxa such as *Hypsilophodon*^48^ which nonetheless shows clear postcranial correlates of cursoriality^37^. Moreover, the floccular fossa houses other structures in addition to the floccular lobe itself, and its size has been found to represent a poor proxy of locomotory mode in extant birds^99^, and likewise does not distinguish quadrupedal and bipedal ornithischians^100^. Consequently, the size of the flocculus appears an unreliable indicator of agility or locomotory behaviour in dinosaurs^99^, necessitating alternative proxies.

The small flocculus in *T. neglectus* contrasts with its extremely elongate anterior semicircular canal (Figs. 2, 4a, d). The semicircular canals sense rotational acceleration of the head and help to coordinate gaze stabilization^7,98^, with elongation (increased radius) of the canals hypothesised to result in greater sensitivity^7,101^. Consequently, measurements of the semicircular canals may provide proxies for locomotory behaviour and agility in extinct organisms (e.g.^7,13,29,98,102^, but see^12,103,104^), and lengthening of the anterior semicircular canal (ASC), and probably also the posterior canal (PSC), which both sense balance (changes in pitch and roll), correlate with bipedality in dinosaurs^102^. Within ornithischians specifically, it has been suggested that the ratio between ASC height: PSC height positively correlates with locomotory agility^24^, based on the observation that the secondary evolution of quadrupedality and reduced agility in ornithopods is accompanied by a reduction in relative ASC height^24^. We find some support for this relationship here by recovering a significant relationship between PSC and skull length but not ASC and skull length, implying that PSC height is controlled by spatial constraints in the skull whereas ASC height varies with ecology. However, this is more likely a result of low statistical power due to the very small taxon sample size available here (n = 10–11), and these results should be considered provisional. Nonetheless, the extremely long ASC suggests acute balance sensitivity, and so possibly high agility, in *Thescelosaurus*.

In sum, synthesis of agility correlates across the skeleton of *Thescelosaurus* yield contradictory signals, with acute balance inferred from the ASC conflicting with the reduced cursoriality of the hindlimb. This conflict may be due to ecological constraints on the hindlimb. *Thescelosaurus* inhabited coastal-plain environments including swamps and marshes^105^, and is more commonly found in channel and near-channel deposits^106,107^. Among large ungulates, semiaquatic taxa that have to travel through slippery or sticky muddy substrates exhibit less cursorial forelimbs, with greater leverage for the muscles powering propulsion^108^. Robust hindlimbs, adapted for stability and powerful retraction, may similarly have been more important for navigation of wet environments than typical cursorial adaptations in *Thescelosaurus*. Moreover, the short PSC (Fig. 4b) and unelongated LSC (Fig. 2)—responsible for sensing turning movements and important during navigation at high speeds^102^^—^further suggests that *T. neglectus* was not highly agile but instead relatively graviportal, and that its acute balance sensitivity does not reflect locomotory performance. Instead, the expanded endosseous labyrinth of *Thescelosaurus*, in conjunction with other endocranial and skeletal data, leads us to alternative hypotheses.

### Semi-fossorial behaviours in *Thescelosaurus* and other small neornithischians

Among vertebrates, the character combination preserved in *T. neglectus* is unique among sampled ornithischians (Fig. 3) but common to many fossorial and semi-fossorial taxa (although anatomical adaptations to fossoriality may differ markedly between clades^109^). Specifically, these are: relatively small overall brain size^110–112^; relatively large olfactory bulbs^112^; limited hearing range, with poor sensitivity to high-frequency sounds (e.g.^113–116^); enhanced equilibrium sensitivity^117^ of the ASC^8^, but not the LSC^118^ or PSC^8^; and more robust skeletal elements with less cursorial limbs^119,120^.

Although the phylogenetic position of *Thescelosaurus* remains controversial^38^, it is broadly considered to be phylogenetically proximate to Orodrominae within Neornithischia (e.g.^22,31,41^), with multiple analyses resolving Orodrominae as the sister-group to Thescelosaurinae, together forming a monophyletic Thescelosauridae (e.g.^27,31,42–46^). Compelling trace^22,89,121^ and body fossil^22,73,122^ evidence for fossorial behaviours are known from the orodromine *Oryctodromeus*, including individuals entombed within preserved subterranean burrows^22,89,121^. Morphological and sedimentological comparison suggests that other orodromine taxa (e.g., *Orodromeus*, *Koreanosaurus*, undescribed Mussentuchit thescelosaurid) were also burrowers^22,97,123,124^. Although *Thescelosaurus* lacks the same degree of anatomical specialization as seen in *Oryctodromeus—*such as the increased sacral count and pubosacral articulations, interpreted as adaptations towards reinforcing the pelvis against forces encountered when bracing the body using the hindlimbs and tail during digging^22,73,122^^—^it does share several morphological characters that have been linked to burrowing in orodromines (Fig. 3b). These include partial fusion of the premaxillae^74^, which may have been used to loosen soil^22^; robust forelimbs^33,73^; and a broad scapula blade^33^ with a strong ventral expansion^34,122^ (note that, although this character is absent in “*T.*” *warreni*^122,125^, this species has since been referred to *Parksosaurus*^31,126^). This expansion of the scapula would have provided greater origination areas for muscle groups (deltoideus scapularis, teres major) important for force generation during manual scratch-digging^22,122^.

Regarding other ecological factors, the relatively large size of *T. neglectus* (up to ~ 4.1 m in total length^31^ and 340 kg in mass^14^, relative to the 20 kg *Orodromeus*^14^), may make burrowing appear unlikely. However, *Oryctrodromeus* individuals up to 3.5 m in length are known from burrow in-fills^89^, and fossilized tunnels have been attributed to substantially larger (up to 1200 kg) mammals^127^. Similarly, wet lowlands, the depositional environment of most *Thescelosaurus* specimens^105–107^, are interpreted by some authors as less suitable for burrowing^128^. However, sediments of the Mussentuchit Member of the Cedar Mountain Formation are notable for being deposited on a tidally influenced coastal plain with periodic saturation^129^, yet taphonomic evidence for burrowing exists in the form of dozens of skeletons of a new, as of yet unnamed species of thescelosaurid^123^. These specimens are interpreted as preserved in subterranean burrows due to their high relative overabundance and unusual levels of articulation compared to other elements of the fauna, and the presence of compacted (~ 1 m), near-complete, multi-individual specimens of multiple age classes^123,130^. Similar factors have been used to support evidence of burrowing in the thescelosaurids *Koreanosaurus*^97^ and *Orodromeus*^124^ in the absence of definitive burrow structures. *Oryctodromeus* is purportedly known from somewhat drier floodplain deposits^22^, although wet coastal deltaic deposits are noted for a large portion of the Blackleaf Formation^131,132^ in which it occurs. Further, many extant animals—including crocodilians^133–135^ and mammals^136,137^^—^do burrow in wet environments, such as riverbanks and waterlogged low-lying areas. In short, periodically waterlogged soils, or riparian environments, do not preclude hypotheses of burrowing in thescelosaurids, and soil saturation may prove to be a limiting factor on burrow preservation, rather than on fossorial behaviour, in these dinosaurs.

**Figure 6:**
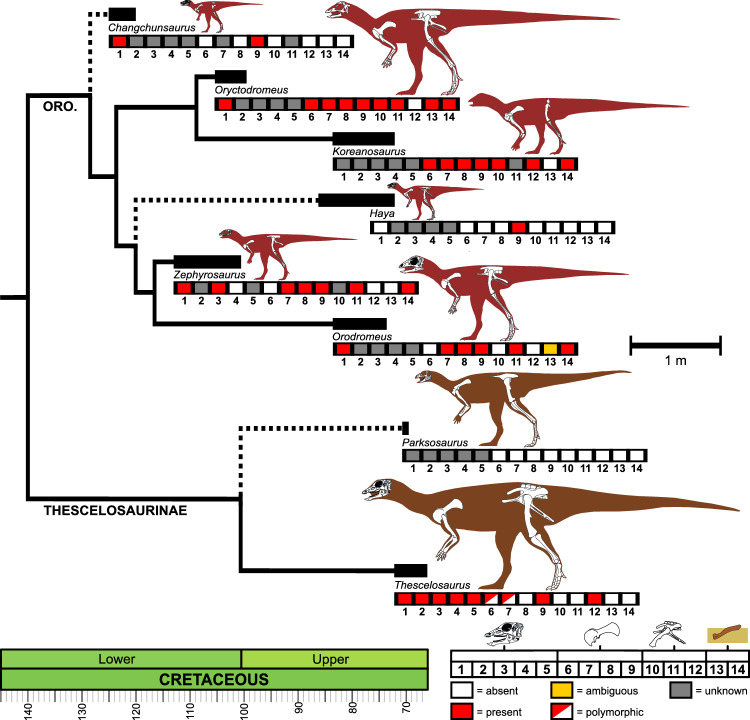
Distribution of characters associated with fossoriality within Thescelosauridae. Simplified time-scaled phylogeny of the Thescelosauridae, after^45,46^, with the positions of taxa of more labile placement in the clade indicated by dotted lines (cf. with^44^). Taxon stratigraphic ranges (see "Methods") indicated by thick lines. Taxon silhouettes and known material from parts of the skeleton bearing discussed characters (skull, pectoral girdle, forelimb, pelvis, hindlimb) are illustrated. Distribution of the following characters and pieces of evidence consistent with fossorial habits (see^22,73,89,97,121,122^ and main text) are indicated. Cranial (1–5): premaxillary fusion (1), reduced EQ (2), large olfactory bulbs (3), enlarged ASC (4), limited hearing range (5). Scapulacoracoid (6–9): fusion of scapula and coracoid (6), well-developed acromion (7), scapular spine (8), prominent posteroventral expansion of scapular blade (9). Pelvis and hindlimb (10–12): seven sacral vertebrae (10), pubosacral articulation (11), reduced cursoriality (12). Occurrence evidence (13–14): body fossils preserved in burrows (13), sedimentological evidence (14). Gross orodromine body shape broadly follows^89,173^, with specific reconstruction and illustrated skeletal anatomy of *Changchunsaurus* following^39^; *Oryctodromeus*^22,89,122^; *Koreanosaurus*^97^, with the holotype and paratype assumed to belong to a single individual after^97^; *Haya*^45^; *Orodromeus*^73,173^; and *Zephyrosaurus*^47^, with postcranial elements reconstructed after those of *Orodromeus*^73,173^. *Parksosaurus* anatomy follows^45^. *Thescelosaurus* is reconstructed primarily from NCSM 15728 but with additional anatomical data and maximum estimated length from^31^. Character coding follows^22,39,45–48,73,74,89,97,121,122^ and discussion in the main text. Oro = Orodrominae. Scale bar for silhouettes = 1 m.

Still, in the absence of any fossilized tunnels or other corroborating ichnological evidence (Fig. 6), the actual extent of fossorial behaviours by *Thescelosaurus* is unclear. The resolution of common ‘fossorial’ traits in *Thescelosaurus* (Fig. 6) indicates that semi-fossorial behaviours may, in fact, be plesiomorphic to Thescelosauridae, or more broadly distributed among Neornithischia in general. This also raises the possibility that the incomplete evidence of fossoriality in *Thescelosaurus* is a result of its divergence from semi-fossorial ancestors: indeed, the unusual character combination and parallelisms with iguanodontian ornithopods^33,36,37^ observed in *Thescelosaurus* may ultimately be explicable through secondary reduction in fossoriality and concomitant increase in body size, although the taxonomic instability of Thescelosaurinae^38^ makes this hypothesis difficult to evaluate. More comprehensive comparison of endocranial and skeletal anatomy across Neornithischia is necessary to further unravel these patterns of ecological evolution through the clade, including evaluation of characters potentially related to digging in other taxa. Nonetheless, taken together, sensorineural and gross morphological lines of evidence support the potential for burrowing behaviours in *Thescelosaurus* itself and/or evolutionary constraints in neurobiology resulting from specializations to a semi-fossorial lifestyle in pre-Maastrichtian thescelosaurids.

Regardless of the extent of fossorial behaviours in *Thescelosaurus*, the observation of endocranial features consistent with fossoriality from a dinosaur clade including known burrowers is significant. These results represent the first neurological specializations to fossoriality identified in any non-avian dinosaur, expanding the range of ecological adaptations recognized in this major clade. Among extant archosaurs, burrowing and denning behaviours are well-known from crocodilians (e.g.^133–135^) and *Apteryx*^138,139^, which each also exhibit high olfactory ratios^56,140^. Olfaction is also important in general surface foraging in these taxa^140–142^, and many birds excavate nesting tunnels (e.g.^143,144^) without obvious morphological specializations, making the extent to which this character can be linked to burrowing in these taxa ambiguous. However, the early development and emphasis of an acute olfactory system may represent a specialization towards subterranean life in burrow-nesting hydrobatid chicks^145^, which navigate^146^ and recognize individuals^146,147^ via olfaction.

The identification of characters consistent with burrowing behaviours in *Thescelosaurus*, from the late Maastrichtian, is further interesting given that the extinction of non-avian dinosaurs across the K-Pg boundary has been attributed to an inability to find shelter^148^ and collapse of primary productivity^149–151^ following the bolide impact at the end of the Cretaceous. During this time, the ability both to shelter from climatic extremes underground and to locate and access hardy, yet buried, resources such as roots and rhizomes would have been critical^148^, and semi-fossorial habits have been suggested as important in the survival of mammalian taxa across this boundary^148,152,153^. The ability of at least some neornithischians to perform these behaviours^22^ and, in particular, resolution of acute olfaction, ability to unearth buried foodstuffs, and possible burrowing capability in the latest Cretaceous *Thescelosaurus*, suggest that such survivorship scenarios may be oversimplified, and more nuanced explanations are necessary to explain the extinction of small-bodied non-avian dinosaurs at the end of the Cretaceous.

## Conclusions

Virtual reconstruction of the endocast of *Thescelosaurus neglectus* reveals a slightly smaller endocast than expected for a reptile of its size and a restricted hearing range, combined with well-developed senses of olfaction and balance. These results contrast with patterns observed in contemporary ornithopods, suggesting that *Thescelosaurus* instead exhibited relatively small group sizes and cognitive abilities within the range of extant reptiles. This character combination, in conjunction with features of the appendicular skeleton, is consistent with burrowing behaviours, as inferred from trace and skeletal fossil evidence from related thescelosaurid taxa. These features may suggest similar semi-fossorial capability in *T. neglectus* or, alternatively, may have been inherited as evolutionary constraints from semi-fossorial ancestors. Indeed, the unusual character combination of *Thescelosaurus* could reflect a secondary reduction in fossoriality and concomitant increase in body size. Either way, these results suggest that semi-fossoriality may have been a general feature of the ecology of thescelosaurids, and potentially neornithischians more generally. Moreover, they provide the first potential neurological specializations to fossoriality identified in a non-avian dinosaur, expanding the range of ecological adaptations recognized within the clade. The identification of potential semi-fossorial capability in the latest Cretaceous *Thescelosaurus* expands our understanding of the ecological niches realized by non-avian dinosaurs and suggests nuance to hypothesized mechanisms explaining their extinction across the end-Cretaceous mass extinction.

## Methods

### Endocranial reconstruction

The skull of NCSM 15728 (‘Willo’), an adult *Thescelosaurus neglectus*, was CT-scanned using a Nikon XTH 225 ST microCT scanner at Duke University, Durham, NC, at a resolution of 87.62 μm. Scan data were then imported into *Avizo* (version 9) for segmentation of separate braincase and skull roof elements. The skull of NCSM 15728 has suffered a mild degree of ventrolateral shearing (Fig. 1a), partially disarticulating the braincase (Fig. 1b, c). In order to repair this damage, the braincase was retrodeformed following a stepwise procedure, as described in^30,154^. To achieve this, the individual elements of the braincase were first isolated, and minor cracks in them repaired, in the *Avizo* segmentation editor. Among the unpaired, midline elements of the braincase, the robust basisphenoid and basioccipital appear not to have suffered plastic deformation. By contrast, the distal tip of the dorsal process of the supraoccipital has been bent laterally; in order to restore symmetry to this element, the distal tip of the supraoccipital was segmented out individually and rotated back into place. The left posterolateral corner of the basioccipital of NCSM 15728 is not associated with the skull but instead in a block containing the postcrania: consequently, it was not scanned. Instead, the right and left halves of the basioccipital were segmented separately, with the right half then being mirrored to yield a symmetrical, composite basioccipital. It should be noted that the occipital condyle of this resulting composite element is still incomplete, but this has no influence on the reconstruction of endocranial tissues.

For each of the paired braincase elements, the better-preserved element was retained. The preservation of each element was judged on evidence of deformation (cracks, warping, asymmetry), topological constraints defined by surrounding elements of the braincase, and comparisons to the osteology of related taxa (e.g.^48^). The left prootic and laterosphenoid are both well-preserved but have become disarticulated: these were moved back into articulation. Whereas the paraoccipital process of the right fused exoccipital and opisthotic is better preserved, the right margin of the foramen magnum has also been squashed medially. Consequently, the better-preserved ventral process of the left exoccipital-opisthotic was mirrored and positioned in place. Shearing of the skull roof has resulted in minor bending of the anterior ends of the frontals and slight deformation to part of their dorsal surface. The less warped left frontal was retained, and these slight deformations were repaired. Shearing has also resulted in crushing of the posterolateral wing of the right parietal: consequently, the left parietal was retained. These elements were then all mirrored to produce symmetrical paired elements.

These retrodeformed elements were all then rearticulated to produce a reconstruction of the undeformed braincase (Fig. 1d). Rearticulation was performed on the basis of the sutural surfaces of each element and topological constraints imposed by surrounding bones. Rearticulation began with the largest and most robust bones (the frontal, parietal, supraoccipital, exoccipital-opisthotic, basioccipital and basisphenoid), helping to constrain the positions of the smaller, and potentially more susceptible to taphonomic deformation and translation, prootics and laterosphenoids. The reconstructed braincase was then tested against three further criteria: its bilateral symmetry, overall dimensional constraints imposed by the rest of the skull, and the continuous alignment of the semi-circular canals within the prootic and supraoccipital. These multiple lines of testing, and the stepwise procedure used herein^154^, are intended to maximise rigour, and minimise biases, in the reconstruction of the original dimensions of the braincase.

The endocranial spaces of the restored braincase were then isolated using the segmentation editor in *Avizo*. This resulted in endocasts of the dural envelope (and, by extension, the brain within) and the semi-circular canals and cochlear duct of the inner ear (Fig. 1e). In addition, the major nerves and blood vessels that drain the brain were reconstructed on the basis of foramina and other osteological correlates on the braincase (e.g.^29,69^). The orbitosphenoids were not ossified in *Thescelosaurus*, as typical for thescelosaurids and early-diverging ornithopods^74^. However, their original ventral extent is inferred to lie at the position of a boss on the anterolateral surface of the laterosphenoids^74^, as observed in some ornithopods^48^. As orbitosphenoids are unknown from phylogenetically proximate taxa, no attempt was made to reconstruct them here. Instead, the position of this boss was used to perform maximum and minimum estimates on the size and curvature of the cerebrum. Comparative measurements of the endocast were made in *Avizo*.

### Endocranial size and reptile encephalization quotient

The total volume of these endocranial reconstructions was measured in *Avizo*, using the ‘Surface Area Volume’ module. The resulting maximum and minimum endocranial volumes of *Thescelosaurus*, excluding the olfactory tract, were used to calculate the Encephalization Quotient^5,6^ (EQ), which compares observed brain volume with that expected from body mass. The non-avian Reptile Encephalization Quotient (REQ) was calculated using the equation of^50^, as follows:

1 $$REQ = M_{Br} /\left( {0.0155*M_{bd}^{0.553} } \right)$$

where *M*_*Br*_ = mass of the brain in grams, and *M*_*bd*_ = body mass, in grams. *M*_*Br*_ is calculated by multiplying the measured volume by a density of 1.036gcm^−3^ for brain tissues^98^. The brain of *Thescelosaurus* was estimated to fill 50% of the endocranial volume, as typical for studies on non-avian dinosaurs^4,6^. Preserved valleculae on the endocranial surfaces of some cerapodan ornithischians^51^ have been used to suggest that the brain filled a larger proportion of the endocranial volume, up to ~ 60%^23,51,88,96^ or even 73% or higher^25^. Although these valleculae were not observed in NCSM 15728 they are known from *Thescelosaurus* assiniboiensis^27^: consequently, a range of REQ values was calculated using fill estimates of both 50% and 60%. Body mass in extinct bipeds can be calculated from the circumference of the femur, employing scaling equations derived from extant taxa^155^. Herein, the mass estimate for a skeletally mature *Thescelosaurus neglectus* of^14^ was employed. This mass estimate was derived from AMNH 5891, a specimen of equal femur length, and similar overall dimensions, to NCSM 15728, and so is expected to provide a reasonable estimate of the mass of this individual.

To place these results in a broader phylogenetic context, they were synthesized with previous measures of REQ from ornithischians. In order to compare these results with those of *T. neglectus*, brain masses were re-calculated from reported endocranial volumes (excluding the olfactory tract^4,5^) assuming a density of 1.036gcm^−3^ for brain tissues^98^. For the sake of comparison, REQs were calculated for estimates of the brain as occupying both 50% and 60% of the endocranial space, although a 60% fill is only likely for some neornithischians (see above). Multiple methods exist to estimate the body mass of extinct taxa, varying from scaling equations through to volumetric models, and different methods may retrieve very different results^156,157^. Previous estimates of ornithischian REQs have employed a combination of these methods, introducing systematic biases into comparisons between them. In an attempt to standardize comparisons between *T. neglectus* and other taxa, previously reported REQs were re-calculated using updated body mass estimates as derived from scaling equations of stylopodial circumferences^14,155,157^ wherever possible. REQs were re-calculated for a specimen of *Psittacosaurus lujiatunensis* (PKUP V1060) using data presented by^158^, but assuming a 50–60% fill of the endocranial spaces by the brain tissues. Similarly, the REQ of *Proa valdearinnoensis* was re-calculated from data from^25^, but using endocranial fill estimates of 50–60%. REQs for specimens of *Iguanodon bernissartensis* (RBINS R51), *Lurdusaurus arenatus* (MNHN GDF 1700) and *Mantellisaurus atherfieldensis* (RBINS R57) were re-calculated using the endocranial volumes reported by^96^ and the body mass estimates calculated for these same specimens by^14^. *Iguanodon* and *Lurdusaurus* were considered quadrupedal after^17,159^, and *Mantellisaurus* as at least facultatively bipedal after^17^, and so the quadrupedal and bipedal mass estimates^14^ were used for these taxa, respectively. Hadrosaurids are considered to have been primarily quadrupedal (e.g.^17^): consequently, only the larger, quadrupedal, mass estimate for *Amurosaurus riabinini* of^88^ was used herein. The REQ of *Kentrosaurus aethiopicus*^6,50^ was also updated using the body mass estimate for a composite skeleton of this taxon calculated by^14^.

The REQ of *Euoplocephalus* was derived from the endocranial volume of AMNH 5337, as calculated by^6^, and the body mass of the similarly-sized and proportioned^160^ AMNH 5404, as calculated by^14^. Similarly, the REQ of *Protoceratops andrewsi* was derived from the endocranial volume of AMNH 6466, a large adult^161^, as calculated by^4^, and the body mass estimate of AMNH 6424, a similarly-sized large adult, of^14^. The endocranial data from *Hypacrosaurus altispinus* used herein comes the reconstruction of ROM 702 by^23^. The body mass of ROM 702 was approximated from the similarly-sized but more complete specimen CMN 8501, following^23^, using the quadrupedal mass estimate of^14^.

The endocranial volume of *Camptosaurus dispar* was calculated by^4^ from YPM VP 1880, a medium-sized individual, approximately two-thirds the length of a large *Camptosaurus*^162^. Consequently, the 400 kg body mass estimate used by^4^ for this specimen, as derived from the scale models of^163^, was retained here as it appears plausible when compared with the 1000–1300 kg estimate calculated from the stylopodial circumferences of a large adult *Camptosaurus* by^14^. The endocranial volumes of *Stegosaurus*, *Edmontosaurus* and *Triceratops* of^4,50^ were derived from specimens lacking sufficient postcranial material from which to derive estimates of body mass. Consequently, to accommodate the range of uncertainty in these taxa, maximum and minimum REQs were calculated from minimum and maximum estimates of body mass, respectively. The minimum body mass estimates were taken from the scale models of^163^, as used in previous estimations of REQ in these taxa^4,50^, whereas the body masses of large individuals of *Stegosaurus ungulatus*, *Edmontosaurus annectens* and *Triceratops horridus*, as calculated from stylopodial circumferences by^14^, were used as maximum body mass estimates. It should be noted that volumetric methods typically retrieve lower body mass estimates for very large taxa than do scaling equations^156,157^: consequently, the maximum REQ estimates for these taxa are almost certainly too large compared to other sampled ornithischians. Nevertheless, as two of these taxa (*Stegosaurus* and *Triceratops*) exhibit two of the lowest REQ values in the sample, the maximum REQ values will represent a conservative estimate of their brain size relative to other taxa.

### Endosseous labyrinth and hearing range

The length of the endosseous cochlear duct was also measured in the *Avizo* viewer. This was then scaled against basicranial length (taken as the length of the basioccipital and basisphenoid, not including the parasphenoid rostrum) and used to calculate the Best Frequency Range (BFR) and Mean Best Hearing (MBH) using the equations of^9^, as follows:

2 $$BFR = \left( {6104.3* ECD} \right) + 6975.2$$

3 $$MBH = \left( {3311.3*ECD} \right) + 4000.8$$

where ECD = Log_10_(scaled endosseous cochlear duct length).

For comparison, the Best Frequency of hearing (BF) and Maximum Frequency (MF) of hearing were also calculated using the equations of^59^, as follows:

4 $$BF = 5.7705e^{ - 0.25*L}$$

5 $$MF = 1.8436*BF + 1.026$$

where *L* = the length of the basilar papilla, in mm. As the length of the basilar papilla is unknown in *Thescelosaurus*, it was estimated as being equal to 2/3rds the length of the endosseous cochlear duct, following^59^. Measurements of the maximum vertical diameter (height) and horizontal diameter (width) of the anterior semicircular canal (ASC) and posterior semicircular canal (PSC), with the labyrinth oriented so the lateral semicircular canal (LSC) lay horizontally, were taken in the *Avizo* viewer. Further, the total length of each of the semicircular canals was also measured as the length of a line drawn through the centre of the lumen of each in three dimensions.

### Phylogenetic tree for comparative paleoneurology

To interpret data from *T. neglectus* in a broader context, an updated version of the informal dinosaurian supertree of^164^ was produced, resulting in a time-scaled species-level topology of 447 taxa (see Supplementary Information for details on tree construction, and Supplementary Data SD1 and SD2 for dated trees). Due to the uncertain phylogenetic position of *Thescelosaurus* two alternative backbone topologies were used for Cerapoda. The first includes *Thescelosaurus* and related taxa as early-diverging ornithopods (e.g.^40,41^), with branching order within Ornithopoda following^41^. The second instead treats *Thescelosaurus*, other thescelosaurines, and orodromines in a monophyletic, non-cerapodan, Thescelosauridae, following^42–44^.

### Olfactory ratio in *Thescelosaurus neglectus* and comparison with other archosaurs

The olfactory ratio^56^ of *T. neglectus* was calculated as the ratio of the longest diameter of the olfactory bulb: longest diameter of the cerebral hemispheres, as measured from the endocast in dorsal view in the *Avizo* viewer. This measurement was taken in two ways, as illustrated in^56^: directly measured from the reconstructed endocast, and also from the maximum width of the fossae for the olfactory bulbs and cerebrum in the skull roof. Both of these methods retrieved identical results. To compare this result to other archosaurs, the olfactory ratio of *T. neglectus* was Log_10_ transformed and combined with the theropod-focused dataset of^56^ (although omitting “*Troodon formosus*” due to the invalidity of that taxon^165^, and taxonomic instability of formerly referred material^166^) and ornithischian-focused dataset of^66^, with additional data on *Erlikosaurus* from^167^. CMN 34825, a subadult^23^ *Corythosaurus* sp., was excluded from this analysis due to its ontogenetic status. In order to estimate a regression line for Dinosauria, *Alligator* data were excluded. Phylogenetic generalized least-squares (pgls) regressions^168^ were then performed between olfactory ratio and body mass as a predictor variable for the remaining sample of dinosaur taxa (n = 25), using the *pgls* function within the ‘caper’ *R*^169^ package^170^, with maximum likelihood estimation of Pagel’s lambda^171^, the phylogenetic signal parameter. Model performance was compared using log likelihoods and the small-sample corrected Aikaike Information Criterion (AICc). The residuals from this regression were then plotted to compare *Thescelosaurus* with other dinosaur taxa. The data used in these analyses is provided in Supplementary Data item SD3, and the full results in SD4.

### Relative vertical semicircular canal development in *Thescelosaurus* and other ornithischians

The relative height of the ASC and PSC has been suggested to correlate with locomotory agility in ornithischians^24^. To compare the height of the vertical semicircular canals across ornithischian taxa, the vertical height (= maximum vertical diameter with the LSC oriented horizontally, see above) of the ASC and PSC of *T. neglectus* were combined with the dataset of^66^ and measurements collected from published digital reconstructions of ornithischian taxa. Pgls regressions were then performed between each of anterior semicircular canal height, posterior canal height, and the ratio between the two as dependent variables, and basal skull length as a predictor variable. All data was Log_10_-transformed prior to analysis. Skull length was preferred for comparison to semicircular canal measurements as head size will be more relevant to their development than total body mass^102^. No attempt was made to calculate head mass due to the lack of data for this attribute in non-avian dinosaurs. The data used in these analyses is provided in Supplementary Data item SD5, and the full results SD6.

### Supplementary Information

Supplementary Information 1.

Supplementary Information 2.

Supplementary Information 3.

Supplementary Information 4.

Supplementary Information 5.

Supplementary Information 6.

Supplementary Information 7.

Supplementary Information 8.

## Data Availability

The trees used for the comparative analyses in this paper are given as Supplementary Data items SD1 and SD2, and the data and results for the pgls regressions in SD3–SD6. The R code for these analyses is provided as Supplementary Data item SD7. The CT scan data and reconstructed surfaces created for this project are available in Morphosource project 000576520 (https://www.morphosource.org/projects/000576520?).
