# Supplementary material for: Neuroanatomy of the late Cretaceous Thescelosaurus neglectus (Neornithischia: Thescelosauridae) reveals novel ecological specialisations within Dinosauria
Source: Sci Rep. 2023 Nov 6;13:19224. doi: 10.1038/s41598-023-45658-3 (PMC10628235; doi:10.1038/s41598-023-45658-3)
Supplement: Supplementary file 1 — Supplementary Information 1. [file 41598_2023_45658_MOESM1_ESM.pdf]

Supplementary information for: Neuroanatomy of the Late Cretaceous *Thescelosaurus neglectus* (Neornithischia: Thescelosauridae) reveals novel ecological specialisations within Dinosauria

David J. Button[1] & Lindsay E. Zanno[2,3]

[1] Bristol Palaeobiology Group, School of Earth Sciences, University of Bristol, UK.

[2]North Carolina Museum of Natural Sciences, Raleigh, North Carolina, USA.

[3]Department of Biological Sciences, North Carolina State University, Raleigh, USA.

Corresponding Author:

David Button

Bristol Palaeobiology Group, School of Earth Sciences, University of Bristol, Bristol, BS8 1TQ, UK.

Email address: david.button@bristol.ac.uk

Institutional abbreviations

AMNH – American Museum of Natural History, New York, USA. CM – Carnegie Museum of Natural History, Pittsburgh, USA. CMN – Canadian Museum of Nature, Ottawa, Canada. FPDM – Fukui Prefectural Dinosaur Museum, Fukui, Japan. FWMSH – Fort Worth Museum of Science and History, Fort Worth, USA. IGM – Mongolian Institute of Geology, Ulaan Bataar, Mongolia. IRSNB – Institut Royal des Sciences Naturelles de Belgique, Brussels, Belgium. MAP – Museo Aragonés de Paleontología (Fundación Conjunto Paleontológico de Teruel-Dinópolis), Teruel, Spain. MB.R. – Museum für Naturkunde, Berlin, Germany. MNHN – Muséum national d'Histoire Naturelle, Paris, France. MPZ – Museo de Ciencias Naturales de la Universidad de Zaragoza, Zaragoza, Spain. NCSM – North Carolina Museum of Natural Sciences, Raleigh, USA. NHMUK – Natural History Museum, London, UK. OMNH – Sam Noble Oklahoma Museum of Natural History, Norman, USA. PKUP – Peking University Palaeontological Collections, Beijing, China. QM – Queensland Museum, Brisbane, Australia. RBINS – Royal Belgian Institute of Natural Sciences, Brussels, Belgium. ROM – Royal Ontario Museum, Toronto, Canada. TMP – Royal Tyrrell Museum of Palaeontology, Drumheller, Canada. UALVP – University of Alberta Laboratory for Vertebrate Paleontology, Edmonton, USA. YPM – Yale Peabody Museum, New Haven, USA.

Endocranial description and comparisons of *T. neglectus*

Gross dural morphology

No valleculae were observed on the endocranial surfaces of *Thescelosaurus*, unlike the condition observed in *T. assiniboiensis*[1] and some other neornithischians[2–4], where it indicates close correspondence between the topology of the endocast surface and that of the underlying brain and other tissues[2]. Nonetheless, the topology of this endocast does provide information regarding the overall size, shape and structure of the brain, cranial nerves and endosseous

labyrinth in *Thescelosaurus*. The retrodeformed braincase reconstruction of *Thescelosaurus neglectus* is illustrated in Figures S1-S2, and the endocast in Figures S2-S3. Comparative measurements are presented in Table S1.

The overall topology of the endocast of *Thescelosaurus neglectus* is broadly similar to that described from other putative thescelosaurids and early-diverging ornithopods[5,6]. It is rostrocaudally elongate, being approximately 40% of the total skull length, similar to those of other neornithischian taxa but unlike the short endocasts of ankylosaurs[7–10]. The cephalic and pontine flexures, separating the fore- and mid-brain and mid- and hind-brain respectively, are both relatively well-developed and roughly equal, as plesiomorphic for dinosaurs[11] and reptiles more generally[12]. This results in a sigmoidal shape more similar to those of thyreophorans[7–10,13], *Pachycephalosaurus*[12], and ceratopsids[14–17] than to either the greater inclination of the cerebellum observed in *Zephyrosaurus, T. assiniboiensis*, early-diverging ornithopods such as *Hypsilophodon*[5] and non-ceratopsid ceratopsians[14,18]; or to the highly reduced flexures and roughly horizontal endocasts of ankylopollexian ornithopods[3,4,19,20].

The anterior extent, overall shape and dorsal morphology of the olfactory tracts are preserved via depressions in the ventral surfaces of the frontals. The olfactory tracts are elongate, with the olfactory bulbs extending far rostral of the cerebral hemispheres, similar to the condition observed in the putative thescelosaurids *Zephyrosaurus* and *T. assiniboiensis*[5]; *Pachycephalosaurus*[11,12] and the ceratopsian *Psittacosaurus*[18]; as well as in non-hadrosaurid ornithopods such as *Dysalotosaurus*[5,6] and, to a lesser extent, *Hypsilophodon*[5], *Iguanodon* and *Mantellisaurus*[4]; but unlike the short olfactory tracts observed in hadrosaurs[3] and thyreophorans[13]. The olfactory lobes are large and diverge rostrally at an angle of ~60°, as typical for ornithischians[3]. A sharp midline crest runs down the dorsal surface of the olfactory tract and the rostral portion of the cerebrum, becoming flush with the dorsal margin of endocast more caudally. This crest probably represents the rostral extension of the longitudinal venous

sinus, as known in other reptiles[12] and reported from some other ornithischians (e.g.[18]). Unfortunately, details of the ventral surface of the olfactory tract cannot be reconstructed due to the absence of ossified presphenoids.

The cerebral hemispheres are distinctly laterally expanded relative to the midbrain, as in other ornithischians[12]. The cerebral hemispheres remain relatively small. At their maximum breadth, the cerebrum is ~1.3 times greater than the width of the olfactory tract, smaller than the ratio observed in hadrosaurs[3] and other ornithopods such as *Dysalotosaurus*[6]. Nonetheless, this ratio in *T. neglectus* is larger than that observed in thyreophorans and marginocephalians[7–13,15,18], or for *Zephyrosaurus* and *T. assiniboiensis*[5], in which the cerebrum breadth is subequal to that of the olfactory tract. The cerebral hemispheres do not bulge strongly dorsally as in iguanodontian ornithopods[3,4,6,19,20]. The ventral anatomy of the cerebrum is difficult to reconstruct due to the absence of ossified orbitosphenoids in *Thescelosaurus*. However, the presence of a rostrolateral boss on the laterosphenoid does allow the original ventral extent of the orbitosphenoids to be estimated[5,21], constraining inferences of the curvature of the cerebrum, and permitting reconstruction of minimum and maximum estimates of its size (see main text). Depending on the reconstruction used, the cerebral hemispheres comprise ~30-33% of the total volume of the endocast (excluding the olfactory bulbs), a higher proportion than previously estimated from the ornithopods *Lurdusaurus* and *Iguanodon*[4], but smaller than in others such as *Proa*[20] and most hadrosaurids[3,22].

The optic lobes can be observed as slight swellings caudolateral to the cerebral hemispheres. The flocculus is similarly indistinct, being apparent only as a small convexity medial to the semicircular canals, similar to those of *Dysalotosaurus*[6] and *T. assiniboiensis*[5], but much smaller than those reconstructed for *Zephyrosaurus*, *Dryosaurus* and *Hypsilophodon*[5] (but see[6]). By contrast, the pituitary fossa, housing both the hypophysis and cavernous venous sinus, is well-developed. Although large, it is relatively narrow and curved caudolaterally,

similar to most other neornithischians and unlike the vertical pituitary fossa typical of thyreophorans[9] and adult *Dysalotosaurus*[6]. Nonetheless, the pituitary fossa of *T. neglectus* appears particularly strongly recurved relative to those of most other ornithischian taxa, comparable only to those of the other thescelosaurids *T. assiniboiensis*, *Zephyrosaurus*[5], and juvenile *Dysalotosaurus*[6]. There is a prominent median dural peak, housed within excavations in the parietals, which may conceivably represent a pineal or parietal organ[5]. However, it should be noted that dural expansions may indicate a variety of structures in archosaurs[23] and the caudal position and vasculature observed in this region (see below) make it more likely that this peak instead corresponded with an extensive dural venous sinus[6,23]. The overall shape and form of the cerebellum and medulla is similar to that of a partial posterior endocast previously reported from a *Thescelosaurus* sp. specimen from the Hell Creek Formation[5], although the fragmentary nature of the latter prevents more detailed comparison.

Cranial nerves

Reconstruction of the braincase allows the exits for many of the cranial nerves to be observed. The optic nerves (CNII) emerged together from a single broad, midline aperture, as typical for dinosaurs[12] and unlike the broadly-separated optic nerves of many ankylosaurs[9]. The absence of ossified orbitosphenoids mean that no information is preserved regarding the exit for the oculomotor (CNIII) nerve. A small foramen partially bound by the laterosphenoid probably represents the exit for the trochlear (CNIV) nerve[21]. The trigeminal nerve (CNV) exited laterally from a large, undifferentiated foramen, indicating that the division of the ophthalmic, maxillary and mandibular rami occurred external to the braincase, as typical for dinosaurs[23]. Still, the path of the ophthalmic ramus (CNV_1) is marked by a deep groove in the lateral surface of the prootic, extending from the anterodorsal corner of the CNV foramen[5,21]. The abducens nerve (CNVI) arises on each side from the rostral end of the medulla, passing into the pituitary

fossa via a narrow canal through the caudal wall of the dorsum sellae. The abducens nerve then exited the pituitary fossa laterally, although this foramen is incompletely preserved. The facial nerve (CNVII) and the acoustic nerve (CNVIII) exit from three small foramina located in a narrow fossa located posteroventral to the trigeminal foramen in the medial surface of the prootic[21]. This is unlike the condition observed in the ornithopods *Tenontosaurus*[24], *Hypsilophodon* and *Dryosaurus*[5,21] where the exit for the posterior acoustic nerve is not housed within this fossa. CNVII exits ventrally, with a narrow, ventrally extending, groove marking the path of the palatine ramus ($CNVII_p$)[5,21]. The acoustic nerve was housed dorsally in this fossa, with the anterodorsally situated anterior ramus ($CNVIII_a$) passing into the utricular recess within the prootic, whereas the posterodorsally situated posterior ramus ($CNVIII_p$) extended into the lagenar recess[21]. In lateral view, the foramen for CNVII is separated from the trigeminal foramen by a prominent crest of bone, which is continuous with the posterior margin of the preotic pendant ventrally.

The foramen metoticum is a small opening set in a deep depression in the lateral surface of the fused exoccipital, bordered anteriodorsally by the crista prootica and separated from the fenestra ovalis by an acute ridge (the crista interfenestrialis). The cranial nerves CNIX-XI, the glossopharyngeal, vagus and accessory nerves exit from this foramen. The vagus nerve, CNX, then curves posteriorly to pass through an anteroposteriorly oriented foramina running through the crista metotica. Behind these are situated the dual foramina for the anterior and posterior rami of the hypoglossal nerve (CNXII). The number of hypoglossal foramina varies within dinosaurs: two separate foramina are also observed in ornithopods[3,5], some thyreophorans[9,25] and *Psittacosaurus*[26]. By contrast, all branches of the hypoglossal nerve canals exited a single foramen in *Pawpawsaurus*[13] and *Jeholosaurus*[27]. Three separate hypoglossal nerve canals are then observed in some ankylosaurids, where the anteriormost branch of CNXII exited into the metotic foramen[8,13]. Similarly, in ceratopsids the large hypoglossal foramina was solely

occupied by the posteriormost hypoglossal nerve canal, with the anteriormost canal(s) exiting with the vagus nerve[15].

Endocranial vasculature

The paired cerebral carotid artery canals enter the pituitary canal caudoventrally as in other dinosaurs (e.g.[6,28]). The canals enter adjacent to one another but then diverge strongly distally, similar to the condition in most ornithischians[3,6,9,17] but unlike the widely separated canals of some ankylosaurs, such as *Pawpawsaurus*[13] and *Talarurus*[10]. The caudal middle cerebral veins are also visible, entering ventrolateral to the dural peak, as typical for dinosaurs (e.g.[9]).

Endosseous labyrinth morphology

In overall form, the endosseous labyrinth resembles those of *T. assiniboiensis*, *Zephyrosaurus* and *Hypsilophodon*[5] and of non-hadrosauriform iguanodontians such as *Dryosaurus*[5], *Dysalotosaurus*[6,29] and *Tenontosaurus*[24]. As in other dinosaurs, the semicircular canals are located caudolateral to the cerebellum, with each being roughly perpendicular with respect to each other. The ASC is narrow and extremely elongate. It is strongly arced, with a maximum height: widest diameter ratio of ~1.52, more similar in shape to those of *T. assiniboiensis*[5], *Dysalotosaurus*[6], *Tenontosaurus*[24], *Psittacosaurus*[18,26], and *Stegoceras*[30] than to the shorter ones of *Zephyrosaurus*[5], *Iguanodon*[31] and *Proa*[20] and, especially, the more square-shaped anterior semicircular canals of eurypodan thyreophorans[9], ceratopsids[17] and hadrosaurs[22]. This results in the height of the ASC being substantially higher than that of the PSC, again dissimilar to the condition in eurypodan thyreophorans[9,13], ceratopsids[15,17], *Zephyrosaurus*[5] and ankylopollexian ornithopods[22], in which the anterior and posterior canals are subequal in height. The height of the ASC also exceeds that of the PSC in *T. assiniboiensis*[5], *Dysalotosaurus*[6], *Tenontosaurus*[24], *Psittacosaurus*[18,26] and *Stegoceras*[30]. However, the ASC of

T. neglectus is even more elongate than in these taxa, with its total length being ~1.5 times that of the other two canals, whereas they are more similar in size in *Dysalotosaurus*[6]. By contrast, the LSC and PSC are approximately equal in length to each other. The ampullae of the ASC and LSC are visible as slight swellings, each approximately equal in size. The ampulla of the PSC is less obvious: however, the PSC is notably thicker than both the ASC and LSC.

The cochlear duct is short, narrow and tapered, similar in both shape and absolute size to non-hadrosaurid ornithopods[6,20,22,24] and ceratopsids[15,17] but dissimilar to the elongated cochleae of hadrosaurs[3,22], *Psittacosaurus*[26] and thyreophorans[9,13], especially ankylosaurids[9,10]. The duct curves slightly medially but does not project strongly anteroventrally, nor is recurved, as in some hadrosaurs[22]. Comparison of the scaled length and width of the cochlear duct to extant taxa shows that *Thescelosaurus* plots most closely to crocodilians (Figure S4) suggesting that, like extant reptiles, *Thescelosaurus* is unlikely to have exhibited complex vocalizations[34].

Overall comparison to Thescelosaurus assiniboiensis *and other ornithischians*

The reconstructed endocast of *T. neglectus* exhibits differences from that of *T. assiniboiensis* including broader cerebral hemispheres, reduced cephalic and pontine flexures, more bifurcated olfactory tract, a more defined dural peak, an expanded pituitary fossa, a more elongate cerebellum, a longer anterior semicircular canal relative to the posterior semicircular canal, and a lengthened cochlear duct (Figure S5). Many of these changes (broadened cerebral hemispheres, reduced cephalic and pontine flexures, increased olfactory bulb size, expansion of the pituitary fossa, elongation of the cerebellum and cochlear duct) are observed through ontogeny in the dryosaurid *Dysalotosaurus*[6], and possibly iguanodontians more generally[6]. The endocast of *T. assiniboiensis* is from a probable juvenile[1], raising the possibility that *Thescelosaurus* shared this ontogenetic trajectory and it was more widely distributed throughout Neornithischia. Still, at present there is insufficient data to fully disentangle

ontogenetic and taxonomic differences between the endocrania of *T. neglectus* and *T. assiniboiensis*.

More broadly, the endocast of *Thescelosaurus* exhibits a combination of characters that are plesiomorphic for Ornithischia (elongate olfactory tract[37], expanded cerebral hemispheres[38]), or at least widely distributed within Dinosauria (expansive dural peak[6,9,39,40]) with an overall shape more similar to those of thyreophorans and ceratopsids than the more flexed endocrania of other thescelosaurids[5], small ornithopods[5,6], and *Psittacosaurus*[18]. These shape differences conform to the allometric relationship plesiomorphic for archosaurs, and broadly conserved within Ornithischia[11,38], and so probably reflect relaxed endocranial spatial constraints in *T. neglectus* due to its greater skull length. Consequently, limited phylogenetic information is provided by the endocast of *T. neglectus*, with it instead differing from other neornithischians primarily in characters related to its sensorineural biology and ecology.

Details on tree construction for comparative analyses

To interpret data from *T. neglectus* in a broader context, additional ornithischian taxa (*Anchiceratops ornatus*, *Arenysaurus ardevoli*, *Kunbarrasaurus ieversi*, *Lurdusaurus arenatus*, *Pachycephalosaurus wyomingensis*, *Pawpawsaurus campbelli*, *Struthiosaurus transylvannicus*, *Talarurus plicatospinus*, *Tarchia teresae*, *Thescelosaurus assiniboiensis* and *Zephyrosaurus schaffi*) were added to a modified version of the informal dinosaurian supertree of[41], resulting in a species-level topology of 445 taxa. Overall ornithischian topology follows[42], stegosaurs[43,44], nodosaurids[45] and ankylosaurids[46,47]: most Mongolian ankylosaurids were collapsed into a polytomy due to uncertainty regarding the putative taxon "*Tarchia teresae*" (cf.[48,49]). Internal ceratopsian topology follows[50–52], pachycephalosaurs[53,54] and iguanodontians[53,55–57]. Due to the uncertain phylogenetic position of *Thescelosaurus*, two alternative backbone topologies were used for Cerapoda. The first includes *Thescelosaurus* and related taxa as early-diverging ornithopods.[42,53,58-60], with branching order within Ornithopoda

following[53] (Supplementary Data S1). The second instead treats *Thescelosaurus* and related taxa such as *Zephyrosaurus* in a monophyletic, non-cerapodan, Thescelosauridae[61-65] (Supplementary Data S2). Relationships of other dinosaur clades remain as described in[41].

These alternative topologies were then time-scaled *a posteriori* via Bayesian tip-dating, performed in MrBayes 3.2.7[66], using the same protocol as in[41]. To do this, the input topologies and an 'empty' character matrix were prepared using the *createMrBayesTipdatingNexus* function in the 'paleotree' version 3.1.3[67] package in the *R* statistical computing environment[68]. A uniform prior was placed on taxon ages, with maximum and minimum ages based upon stratigraphic occurrences, as extracted from the Paleobiology Database (https://paleobiodb.org) using the following parameters: taxonomy = Dinosauria, taxonomic resolution = species-level, interval = Triassic-Cretaceous; download was performed in April 2017, subsequently updated with data for *Kunbarrasaurus ieversi* and *Tarchia teresae*, downloaded in January 2021). Further, a uniform prior was also placed on the root age of Dinosauria, with a maxmimum and minimum bounds of 249 Ma and 237 Ma, respectively, set based upon[69]. Default priors assigned by *createMrBayesTipdatingNexus* (speciation rate = exp(1); extinction rate = beta[1, 1]; fossilization rate = beta[1, 1]; sampling strategy = random. Clock priors: clock rate = normal(0.0025, 0.1); clock variance = igr; igr variance = a 'vague' prior: uniform(0.0001, 200) after[70]) were used for other parameters. For both of the alternative cerapod backbone topologies the analysis was run four times over 20000000 generations, sampling every 1000, with the first 50% of trees discarded as burn-in, and with random resolution of polytomies. The posterior sample of resolved, dated trees for cerapod backbone topology 1 are given in Supplementary Data SD1; those for cerapod backbone topology 2 are given in Supplementary Data SD2. Fifty trees were drawn randomly from the posterior samples of both the alternative cerapod backbone topologies. Downstream analyses were performed across this sample of 100 trees, in order to accommodate uncertainty in stratigraphic age and phylogenetic resolution of taxa.

Supplementary figures

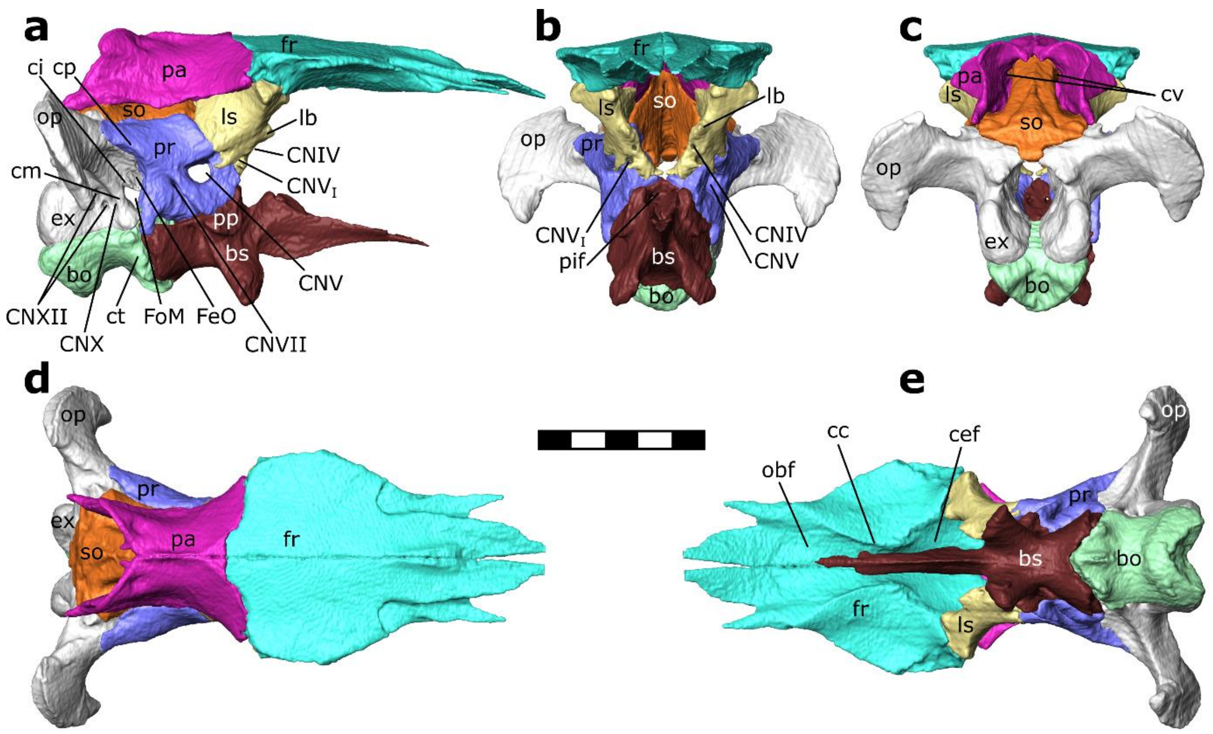

Figure S1: The braincase reconstruction of *T. neglectus*. Retrodeformed braincase in right lateral (a), rostral (b), caudal (c), dorsal (d), and ventral (e) views. Scale bar = 50 mm. Abbreviations: bo = basioccipital, bs = parabasisphenoid, cc = crista cranii, cef = impression left by cerebral hemispheres, ci = crista interfenestrialis, cm = crista metotica, cp = crista prootica, ct = crista tuberalis, cv = caudal middle cerebral vein, ex = exoccipital, FeO = fenestra ovalis, fr = frontal, FoM = foramen metotica, lb = boss on laterosphenoid, ls = laterosphenoid, obf = impression left by olfactory bulb, pa = parietal, pif = pituitary fossa, pp = preotic pendant, so = supraoccipital. Nerve exits: CNIV = exit for the trochlear nerve, CNV = exit for the trigeminal nerve, CNV_I = groove for the ophthalmic ramus of the trigeminal nerve, CNVII = exit for the facial nerve, CNX = exit for the vagus nerve, CNXII = exits for the hypoglossal nerve.

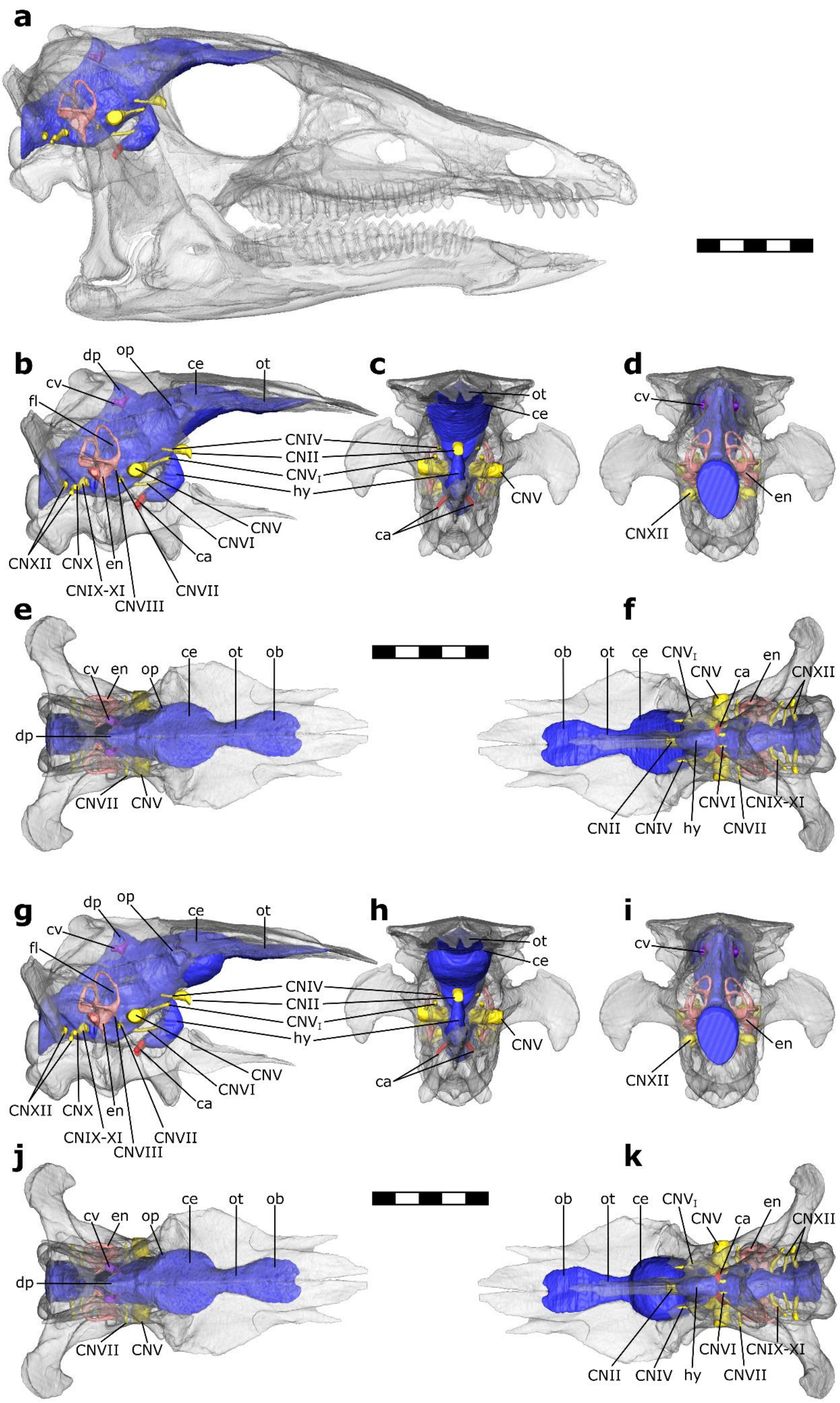

a
b
dp
op
ce
ot
cv
fl
CNIV
CNII
CNV$_I$
hy
CNV
CNVI
ca
CNXII
CNX
en
CNIX-XI
CNVII
CNVIII
c
ot
ce
CNV
ca
d
cv
en
CNXII
e
cv
en
op
ce
ot
ob
dp
CNVII
CNV
f
ob
ot
ce
CNV$_I$
CNV
ca
en
CNXII
CNII
CNIV
hy
CNVI
CNVII
CNIX-XI
g
dp
op
ce
ot
cv
fl
CNIV
CNII
CNV$_I$
hy
CNV
CNVI
ca
CNXII
CNX
en
CNIX-XI
CNVIII
CNVII
h
ot
ce
CNV
ca
i
cv
en
CNXII
j
cv
en
op
ce
ot
ob
dp
CNVII
CNV
k
ob
ot
ce
CNV$_I$
CNV
ca
en
CNXII
CNII
CNIV
hy
CNVI
CNIX-XI
CNVII

Figure S2: The reconstructed skull, braincase, and endocranial tissues of *T. neglectus*. a-f) Bones rendered as translucent and endocranial tissues as solid for the minimum (a-f) and maximum (g-k) reconstructions of cerebrum size. a) Retrodeformed skull in right lateral view. b-k) braincase in right lateral (b, g), rostral (c, h), caudal (d, i), dorsal (e, j), and ventral (f, k) views. Scale bars = 50 mm. Abbreviations: ca = carotid artery, ce = cerebral hemispheres, cv = caudal middle cerebral vein, dp = dural peak, en = endosseous labyrinth, fl = flocculus, hy = hypophysis, op = optic lobe, ot = olfactory tract. Nerves: CNII = optic nerves, CNIV = trochlear nerve, CNV = trigeminal nerve, CNV_I = ophthalmic ramus of the trigeminal nerve, CNVII = facial nerve, CNVIII = acoustic nerve, CNIX = glossopharyngeal nerve, CNX = vagus nerve, CNXI = accessory nerve, CNXII = hypoglossal nerve.

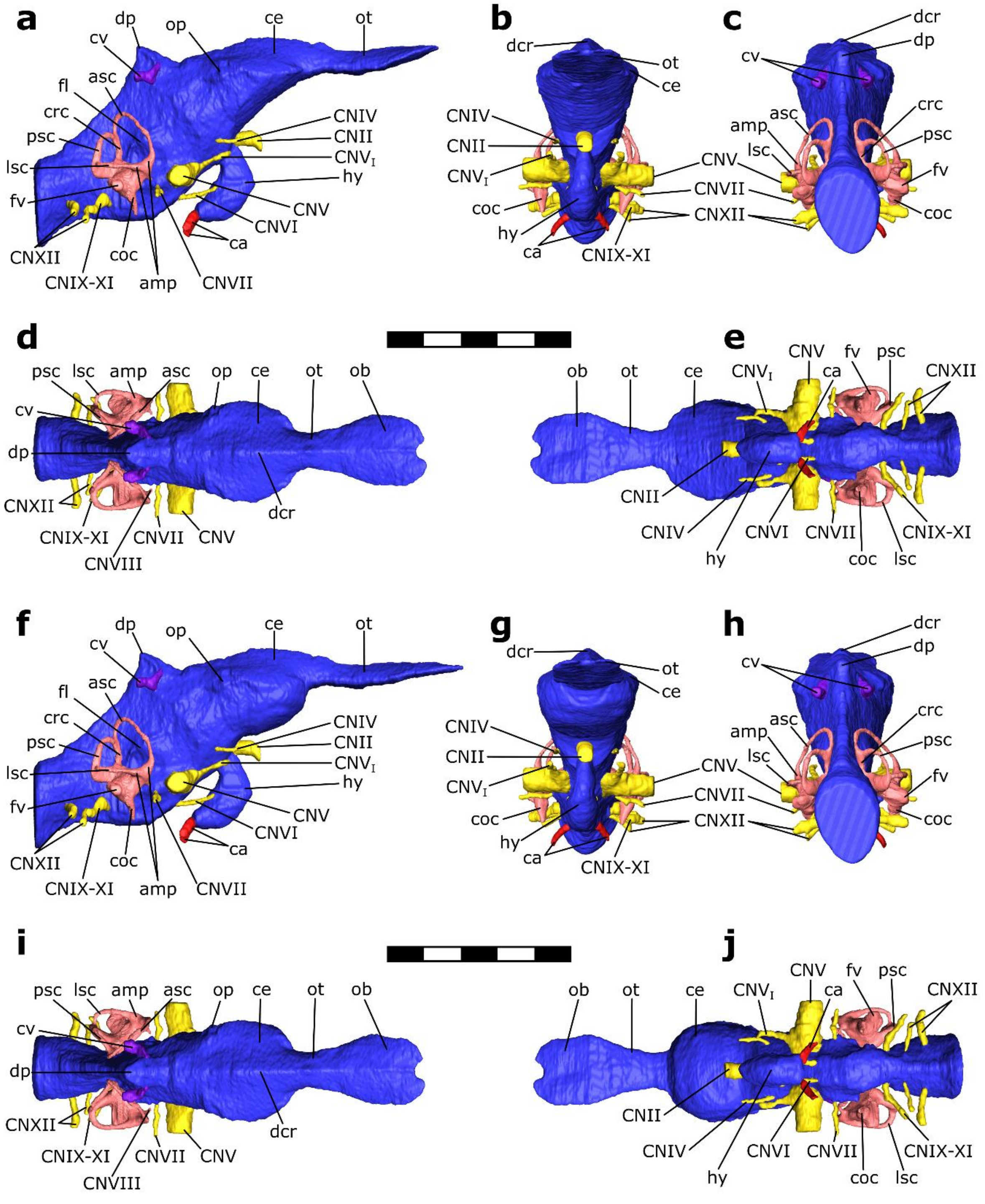
a
dp
op
ce
ot
cv
asc
fl
crc
psc
lsc
fv
CNXII
CNIX-XI
coc
amp
CNVII
CNIV
CNII
CNV$_{I}$
hy
CNV
CNVI
ca
b
dcr
ot
ce
CNIV
CNII
CNV$_{I}$
coc
hy
ca
CNIX-XI
c
dcr
dp
cv
amp
asc
lsc
crc
psc
CNV
CNVII
CNXII
fv
coc
d
psc
lsc
amp
asc
op
ce
ot
ob
cv
dp
CNXII
CNIX-XI
CNVIII
CNVII
CNV
dcr
e
ob
ot
ce
CNV$_{I}$
CNV
ca
fv
psc
CNXII
CNII
CNIV
hy
CNVI
CNVII
coc
lsc
CNIX-XI
f
dp
op
ce
ot
cv
asc
fl
crc
psc
lsc
fv
CNXII
CNIX-XI
coc
amp
CNVII
CNIV
CNII
CNV$_{I}$
hy
CNV
CNVI
ca
g
dcr
ot
ce
CNIV
CNII
CNV$_{I}$
coc
hy
ca
CNIX-XI
h
dcr
dp
cv
amp
asc
lsc
crc
psc
CNV
CNVII
CNXII
fv
coc
i
psc
lsc
amp
asc
op
ce
ot
ob
cv
dp
CNXII
CNIX-XI
CNVIII
CNVII
CNV
dcr
j
ob
ot
ce
CNV$_{I}$
CNV
ca
fv
psc
CNXII
CNII
CNIV
hy
CNVI
CNVII
coc
lsc
CNIX-XI

Figure S3: Reconstructed endocast of *T. neglectus*. Results depicted for the minimum (a-e) and maximum (f-j) reconstructions of cerebrum size in right lateral (a, f), rostral (b, g), caudal (c, h), dorsal (i), and ventral (j) views. Scale bars = 50 mm. Abbreviations: ca = carotid artery, ce = cerebral hemispheres, cv = caudal middle cerebral vein, dcr = dorsal crest (= longitudinal venous sinus?), dp = dural peak, fl = flocculus, hy = hypophysis, op = optic lobe, ot = olfactory tract. Nerves: CNII = optic nerves, CNIV = trochlear nerve, CNV = trigeminal nerve, CNV_I = ophthalmic ramus of the trigeminal nerve, CNVII = facial nerve, CNVIII = acoustic nerve, CNIX = glossopharyngeal nerve, CNX = vagus nerve, CNXI = accessory nerve, CNXII = hypoglossal nerve. Endosseous labyrinth: ASC = anterior semicircular canal, LSC = lateral semicircular canal, PSC = posterior semicircular canal, amp = ampulla, crc = crus communis, coc = cochlear duct, fv = vestibule of inner ear.

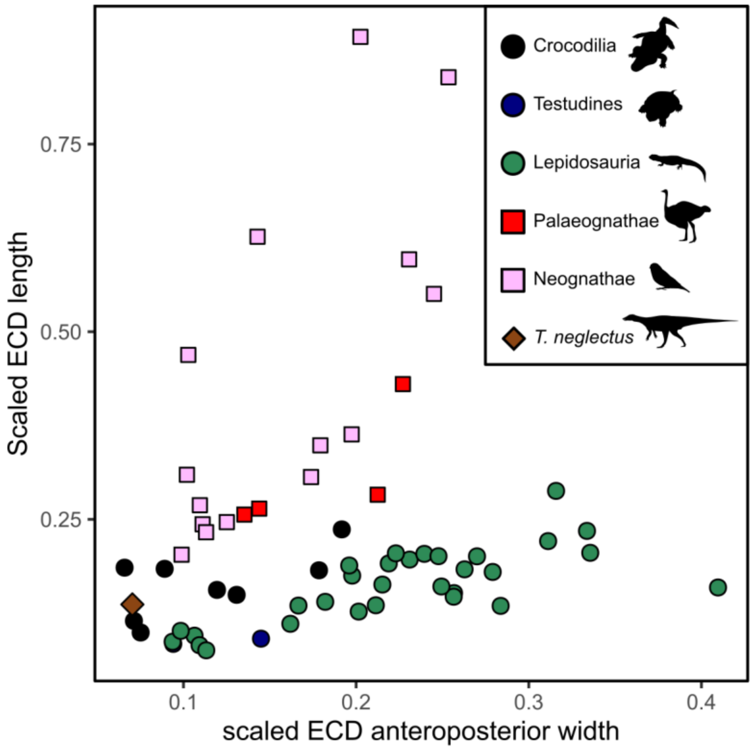

Figure S4: Comparison of Endosseous Cochlear Duct (ECD) dimensions of *Thescelosaurus neglectus* with those of some extant sauropsid taxa. Scaled ECD length plotted against scaled ECD width following[34], including data presented herein for *T. neglectus*.

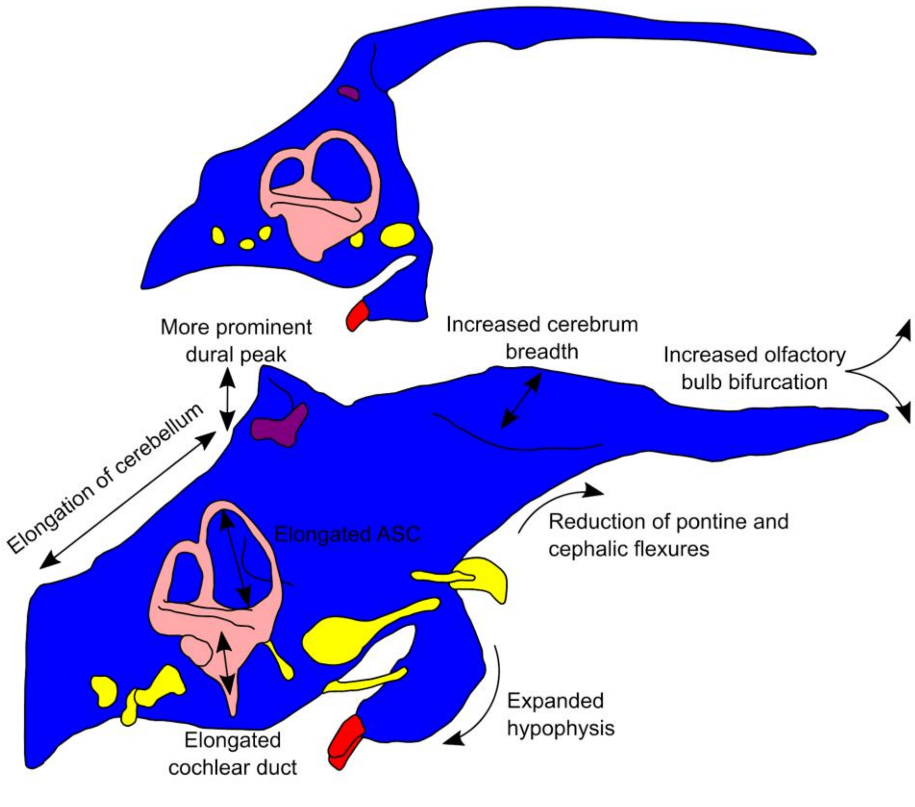

Figure S5. Comparison of the endocasts of *Thescelosaurus assiniboiensis* and *Thescelosaurus neglectus*. Endocast of *T. assiniboiensis* (top) redrawn after (Galton, 1989). Endocast of *T. neglectus* (bottom) based upon results from NCSM 15728 presented herein. Observed shape differences between the endocrania of these taxa are noted on the endocast of *T. neglectus*. ASC = anterior semicircular canal.

Supplementary Tables

Table S1: Endocranial measurements from NCSM 15728. Volumetric measurements are given for both minimum and maximum estimates of cerebrum size (see Materials & Methods). Measured cerebrum volumes do not include the olfactory tract. Total semicircular length was measured along a line drawn through the center of the lumen of each canal in 3D. Anterior and posterior semicircular canal heights measured as the vertical diameter when the lateral semicircular canal is oriented horizontally in lateral view. ASC = anterior semicircular canal, LSC = lateral semicircular canal, PSC = posterior semicircular canal.

**Measurements from the reconstructed dural envelope of NCSM15728**	
Total endocast length /mm	117
Olfactory tract length /mm	39
Cerebral hemispheres length /mm	24.7
Cerebral hemispheres width /mm	27.5
Cephalic flexure /°	151
Pontine flexure /°	150
Olfactory tract volume /mm^3	1822.1
Olfactory bulb volume /mm^3	959.6
Minimum cerebrum volume (not including olfactory tract) /mm^3	8204.95
Maximum cerebrum volume (not including olfactory tract) /mm^3	9568.55
Minimum total endocast volume /mm^3	29071.1
Maximum total endocast volume /mm^3	30434.7
**Measurements from the endosseous labyrinth of NCSM 15728**	
Total ASC length /mm	42.8
ASC height/mm	15
Total LSC length /mm	28.27
Total PSC length /mm	30.87
PSC height /mm	9.1
Endosseous cochlear duct length /mm	10
Endosseous cochlear duct anteroposterior length /mm	3.5

Table S2: Comparison of the olfactory ratio of *T. neglectus* with other ornithischians. [a] = body mass estimate from[32], other mass estimates derived from the references in the source column. Specimens from adult individuals unless otherwise indicated.

Taxon	Specimen	OR	Body mass /kg	Source
Thescelosaurus neglectus	NCSM 15728	0.69	338.5[a]	This study.
Stegosaurus stenops	CM 106	0.64	4722.8[a]	[9]
Euoplocephalus tutus	AMNH 5404	0.729	2329.6[a]	[9, 33]
Corythosaurus sp.	CMN 34825 (subadult)	0.684	3078.5	[3, 17]
Hypacrosaurus altispinus	ROM 702	0.517	2478	[3, 17]
Stegoceras validum	UALVP 2	0.40	26.7	[17, 30]
Triceratops sp.	FPDM-V-9677	0.401	4963.6	[17]

Table S3: Comparison of calculated auditory sensitivity of *T. neglectus* with values previously reported from other ornithischians and selected theropods. MBH = frequency of Mean Best Hearing, as calculated from the equation of[34]; BHR = Best Hearing Range, as calculated from the equation of[34]; FBH = Frequency of Beast Hearing, as calculated from the equation of[35]; ULH = Upper Limit of Hearing, as calculated from the equation of[35]. Results for *T. neglectus* were calculated using measured basicranial length (69 mm: taken as the length of the basioccipital and basisphenoid, excludes the parasphenoid rostrum, measured from the retrodeformed braincase in lateral view, parallel to the long axis of the cranial vault). Results for *Kunburrasaurus*, *Pawpawsaurus*, *Lambeosaurus*, *Corythosaurus*, *Hypacrosaurus*, *Pachyrhinosaurus*, *Triceratops* and *Tyrannosaurus* re-calculated from basilar papilla lengths estimated by[17]. Specimens from adult individuals unless otherwise stated.

Taxon	Specimen	MBH /Hz	BHR /Hz	FBH /Hz	ULH /Hz	Source
Thescelosaurus neglectus	NCSM 15728	1223.12	1854.61	1089.82	3051.78	This study
Kunburrasaurus ieversi	QM F18101	-	-	101	1228	9, 17
Pawpawsaurus campbelli	FWMSH 93B.00026	-	-	243	1491	13, 17
Dysalotosaurus lettwvorbecki	MB.R.1367; MB.R.1370; MB.R.1372; MB.R. 1373 (subadult)	2100	3500	-	-	29
Lambeosaurus sp.	ROM 758 (juvenile)	-	-	1245	3339	3, 17
Corythosaurus sp.	ROM 759 (juvenile)	-	-	794	2507	3, 17
Corythosaurus sp.	CMN 34825 (subadult)	-	-	743	2412	3, 17
Hypacrosaurus altispinus	ROM 702	-	-	357	1700	3, 17
Pachyrhinosaurus lakustai	TMP 1989.55.1243	-	-	458	1887	15, 17
Triceratops sp.	FPDM-V-9775	-	-	290	1577	17
Tyrannosaurus rex	AMNH FR 5117	-	-	579	2109	17, 23
Erlikosaurus andrewsi	IGM 100/111	-	-	910-1600	2700-4000	28
Velociraptor mongoliensis	IGM 100/976	2368	3965	-	-	36

Table S4: Comparison between the relative volume of the cerebral hemispheres of *T. neglectus* and ornithopods. Relative volume was calculated as the cerebrum (excluding the olfactory tract) divided by the total volume of the endocast (excluding the olfactory tract). All specimens inferred to belong to adult individuals unless otherwise indicated. [a] = calculated as total forebrain volume divided by total endocast volume (both excluding the olfactory tract and hypophysis).

Taxon	Specimen	Relative cerebral hemispheres volume (%)	Source
Thescelosaurus neglectus	NCSM 15728	30.1-33.4	This study
Dysalotosaurus lettwvorbecki	MB.R. 1370, 1372, 1377, 1378	~16[a]	[20]
Lurdusaurus arenatus	MNHN GDF 1700	19	[4]
Proa valdearinnoensis	MAP AR-1-2012	39[a]	[20]
Iguanodon bernissartensis	RBINS R51	19	[4]
Amurosaurus riabinini	IRSNB R 279	30	[19]
Arenysaurus ardevoli	MPZ 2008/1 (subadult - adult)	53.27	[22]
Lambeosaurus sp.	ROM 758 (juvenile)	38.5	[3]
Corythosaurus sp.	ROM 759 (juvenile)	45.4	[3]
Corythosaurus sp.	ROM 34825 (subadult)	38.1	[3]
Hypacrosaurus altispinus	ROM 702	42.6	[3]

References

1. Brown, C. M., Boyd, C. A. & Russell, A. P. A new basal ornithopod dinosaur (Frenchman Formation, Saskatchewan, Canada), and implications for late Maastrichtian ornithischian diversity in North America. *Zool. J. Linn. Soc.* **163**, 1157–1198 (2011).
2. Evans, D. C. New evidence on brain−endocranial cavity relationships in ornithischian dinosaurs. *Acta Palaeontol. Pol.* **50**, 617–622 (2005).
3. Evans, D. C., Ridgely, R. & Witmer, L. M. Endocranial anatomy of lambeosaurine hadrosaurids (Dinosauria: Ornithischia): A sensorineural perspective on cranial crest function. *Anat. Rec.* **292**, 1315–1337 (2009).
4. Lauters, P., Coudyzer, W., Vercauteren, M. & Godefroit, P. The brain of *Iguanodon* and *Mantellisaurus*: Perspectives on ornithopod evolution. in: *Bernissart Dinosaurs in depth: A window on Early Cretaceous Terrestrial Ecosystems* (ed. Godefroit, P.) 213–224 (Indiana University Press, 2012).
5. Galton, P. M. Crania and endocranial casts from ornithopod dinosaurs of the families Dryosauridae and Hypsilophodontidae (Reptilia; Ornithischia). *Geol. Palaeontol.* **23**, 217–239 (1989).
6. Lautenschlager, S. & Hübner, T. Ontogenetic trajectories in the ornithischian endocranium. *J. Evol. Biol.* **26**, 2044–2050 (2013).
7. Witmer, L. M. & Ridgely, R. C. The paranasal air sinuses of predatory and armored dinosaurs (Archosauria: Theropoda and Ankylosauria) and their contribution to cephalic structure. *Anat. Rec.* **291**, 1362–1388 (2008).
8. Miyashita, T., Arbour, V. M., Witmer, L. M. & Currie, P. J. The internal cranial morphology of an armoured dinosaur *Euoplocephalus* corroborated by X-ray computed tomographic reconstruction. *J. Anat.* **219**, 661–675 (2011).
9. Leahey, L. G., Molnar, R. E., Carpenter, K., Witmer, L. M. & Salisbury, S. W. Cranial

osteology of the ankylosaurian dinosaur formerly known as *Minmi* sp. (Ornithischia: Thyreophora) from the Lower Cretaceous Allaru Mudstone of Richmond, Queensland, Australia. *PeerJ* **3**, e1475 (2015).

10. Paulina-Carabajal, A., Lee, Y. N., Kobayashi, Y., Lee, H. J. & Currie, P. J. Neuroanatomy of the ankylosaurid dinosaurs *Tarchia teresae* and *Talarurus plicatospineus* from the Upper Cretaceous of Mongolia, with comments on endocranial variability among ankylosaurs. *Palaeogeogr. Palaeoclimatol. Palaeoecol.* **494**, 135–146 (2018).

11. Griffin, E. B. Pachycephalosaur paleoneurolgy (Archosauria : Ornithischia). *J. Vertebr. Paleontol.* **9**, 67–77 (1989).

12. Hopson, J. A. Paleoneurology. in *Biology of the Reptilia, vol. 9* (eds. Gans, C., Northcutt, R. H. & P., U.) 39–146 (Academic Press, 1979).

13. Paulina-Carabajal, A., Lee, Y.-N. & Jacobs, L. L. Endocranial morphology of the primitive nodosaurid dinosaur *Pawpawsaurus campbelli* from the Early Cretaceous of North America. *PLoS One* **11**, e0150845 (2016).

14. Brown, B. & Schlaikjer, E. M. The structure and relationships of *Protoceratops*. *Ann. N. Y. Acad. Sci.* **40**, 133–206 (1940).

15. Witmer, L. M. & Ridgely, R. C. Structure of the brain cavity and inner ear of the centrosaurine ceratopsid dinosaur *Pachyrhinosaurus* based on CT scanning and 3D visualization. in *A new horned dinosaur from an Upper Cretaceous bone bed in Alberta* (eds. Currie, P. J., Langston, W. & Tanke, D. H.) 117-144 (National Research Council of Canada Monograph Publishing Program, 2008).

16. Forster, C. A. New information on the skull of *Triceratops*. *J. Vertebr. Paleontol.* **16**, 246–258 (1996).

17. Sakagami, R. & Kawabe, S. Endocranial anatomy of the ceratopsid dinosaur *Triceratops*

and interpretations of sensory and motor function . *PeerJ* **8**, e9888 (2020).

18. Zhou, C. F., Gao, K. Q., Fox, R. C. & Du, X. K. Endocranial morphology of psittacosaurs (Dinosauria: Ceratopsia) based on CT scans of new fossils from the Lower Cretaceous, China. *Palaeoworld* **16**, 285–293 (2007).

19. Lauters, P., Vercauteren, M., Bolotsky, Y. L. & Godefroit, P. Cranial endocast of the lambeosaurine hadrosaurid *Amurosaurus riabinini* from the Amur Region, Russia. *PLoS One* **8**, e78899 (2013).

20. Knoll, F. *et al.* Palaeoneurology of the early cretaceous iguanodont *Proa valdearinnoensis* and its bearing on the parallel developments of cognitive abilities in theropod and ornithopod dinosaurs. *J. Comp. Neurol.* **529**, 3922-3945 (2021).

21. Boyd, C. A. The cranial anatomy of the neornithischian dinosaur *Thescelosaurus neglectus*. *PeerJ* **2**, e669 (2014).

22. Cruzado-Caballero, P., Fortuny, J., Llacer, S. & Canudo, J. Paleoneuroanatomy of the European lambeosaurine dinosaur *Arenysaurus ardevoli*. *PeerJ* **3**, e802 (2015).

23. Witmer, L. M., Ridgely, R. C., Dufeau, D. L. & Semones, M. C. Using CT to Peer into the Past: 3D Visualization of the Brain and Ear Regions of Birds, Crocodiles, and Nonavian Dinosaurs. in *Anatomical Imaging* (eds. Endo, H. & Frey, R.) 67–87 (Springer Japan, 2009).

24. Thomas, D. The cranial anatomy of *Tenontosaurus tilletti* Ostrom, 1970 (Dinosauria, Ornithopoda). *Palaeontol. Electron.* **18,** 37A (2015).

25. Galton, P. M. Skull bones and endocranial casts of stegosaurian dinosaur *Kentrosaurus* Hennig, 1915 from Upper Jurassic of Tanzania, East Africa. *Geol. Palaeontol.* **22**, 123–143 (1988).

26. Bullar, C. M., Zhao, Q., Benton, M. J. & Ryan, M. J. Ontogenetic braincase development in *Psittacosaurus lujiatunensis* (Dinosauria: Ceratopsia) using micro-computed

tomography. *PeerJ* **7**, e7217 (2019).

27. Barrett, P. M. & Han, F.-L. Cranial anatomy of *Jeholosaurus shangyuanensis* (Dinosauria: Ornithischia) from the Early Cretaceous of China. *Zootaxa* **55**, 31–55 (2009).

28. Lautenschlager, S., Rayfield, E. J., Altangerel, P., Zanno, L. E. & Witmer, L. M. The Endocranial anatomy of Therizinosauria and its implications for sensory and cognitive function. *PLoS One* **7**, e52289 (2012).

29. Sobral, G., Hipsley, C. A. & Müller, J. Braincase redescription of *Dysalotosaurus lettowvorbecki* (Dinosauria, Ornithopoda) based on computed tomography. *J. Vertebr. Paleontol.* **32**, 1090–1102 (2012).

30. Bourke, J. M. *et al.* Breathing life into dinosaurs: Tackling challenges of soft-tissue restoration and nasal airflow in extinct species. *Anat. Rec.* **297**, 2148–2186 (2014).

31. Norman, D. B. Basal Iguanodontia. in *The Dinosauria* (eds. Weishampel, D. B., Dodson, P. & Osmolska, H.) 413–487 (University of California Press, 2004).

32. Benson, R. B. J. et al. Rates of dinosaur body mass evolution indicate 170 million years of sustained ecological innovation on the avian stem lineage. *PLoS Biol.* **12**, e1001853 (2014).

33. Arbour, V. M. & Currie, P. J. *Euoplocephalus tutus* and the diversity of ankylosaurid dinosaurs in the Late Cretaceous of Alberta, Canada, and Montana, USA. *PLoS One* **8**, e62421 (2013).

34. Walsh, S. A., Barrett, P. M., Milner, A. C., Manley, G. & Witmer, L. M. Inner ear anatomy is a proxy for deducing auditory capability and behaviour in reptiles and birds. *Proc. R. Soc. B Biol. Sci.* **276**, 1355–1360 (2009)

35. Gleich, O., Dooling, R. J. & Manley, G. A. Audiogram, body mass, and basilar papilla length: Correlations in birds and predictions for extinct archosaurs. *Naturwissenschaften*

92, 595–598 (2005).

36. King, J. L., Sipla, J. S., Georgi, J. A., Balanoff, A. M. & Neenan, J. M. The endocranium and trophic ecology of Velociraptor mongoliensis. J. Anat. 237, 861–869 (2020).

37. Holloway, W. L., Claeson, K. M. & Okeefe, F. R. A virtual phytosaur endocast and its implications for sensory system evolution in archosaurs. *J. Vertebr. Paleontol.* **33**, 848–857 (2013).

38. Hopson, J. A. Paleoneurology. in *Biology of the Reptilia, vol. 9* (eds. Gans, C., Northcutt, R. H. & P., U.) 39–146 (Academic Press, 1979).

39. Sampson, S. D. & Witmer, L. M. Craniofacial anatomy of *Majungasaurus crenatissimus* (Theropoda: Abelisauridae) from the Late Cretaceous of Madagascar. *J. Vertebr. Paleontol.* **27**, 32–104 (2007).

40. Knoll, F., Witmer, L. M., Ortega, F., Ridgely, R. C. & Schwarz-Wings, D. The braincase of the basal sauropod dinosaur *Spinophorosaurus* and 3D reconstructions of the cranial endocast and inner ear. *PLoS One* **7**, e30060 (2012).

41. Button, D. J. & Zanno, L. E. Repeated evolution of divergent modes of herbivory in non-avian dinosaurs. *Curr. Biol.* **30**, 158-168.e4 (2020).

42. Butler, R. J., Upchurch, P. & Norman, D. B. The phylogeny of the ornithischian dinosaurs. *J. Syst. Palaeontol.* **6**, 1–40 (2008).

43. Raven, T. J. & Maidment, S. C. R. A new phylogeny of Stegosauria (Dinosauria, Ornithischia). *Palaeontology* **60**, 401–408 (2017).

44. Raven, T. J. & Maidment, S. C. R. The systematic position of the enigmatic thyreophoran dinosaur *Paranthodon africanus*, and the use of basal exemplifiers in phylogenetic analysis. *PeerJ* **6**, e4529 (2018).

45. Thompson, R. S., Parish, J. C., Maidment, S. C. R. & Barrett, P. M. Phylogeny of the ankylosaurian dinosaurs (Ornithischia: Thyreophora). *J. Syst. Palaeontol.* **10**, 301–312

(2012).

46. Arbour, V. M. & Currie, P. J. Systematics, phylogeny and palaeobiogeography of the ankylosaurid dinosaurs. *J. Syst. Palaeontol.* **14**, 385–444 (2016).

47. Arbour, V. M. & Evans, D. C. A new ankylosaurine dinosaur from the Judith River Formation of Montana, USA, based on an exceptional skeleton with soft tissue preservation. *R. Soc. Open Sci.* **4**, 161086 (2017).

48. Arbour, V. M., Currie, P. J. & Badamgarav, D. The ankylosaurid dinosaurs of the Upper Cretaceous Baruungoyot and Nemegt formations of Mongolia. *Zool. J. Linn. Soc.* **172**, 631–652 (2014).

49. Penkalski, P. & Tumanova, T. The cranial morphology and taxonomic status of *Tarchia* (Dinosauria: Ankylosauridae) from the Upper Cretaceous of Mongolia. *Cretac. Res.* **70**, 117–127 (2017).

50. Farke, A. A. *et al.* A new centrosaurine from the late cretaceous of alberta, canada, and the evolution of parietal ornamentation in horned dinosaurs. *Acta Palaeontol. Pol.* **56**, 691–702 (2011).

51. Brown, C. M. & Henderson, D. M. A new horned dinosaur reveals convergent evolution in cranial ornamentation in Ceratopsidae. *Curr. Biol.* **25**, 1641–1648 (2015).

52. Morschhauser, E. M., You, H., Li, D. & Dodson, P. Phylogenetic history of *Auroraceratops rugosus* (Ceratopsia: Ornithischia) from the Lower Cretaceous of Gansu Province, China. *J. Vertebr. Paleontol.* **38**, 117–147 (2018).

53. Dieudonné, P. E., Cruzado-Caballero, P., Godefroit, P. & Tortosa, T. A new phylogeny of cerapodan dinosaurs. *Hist. Biol.* **10**, 2335-2355 (2021).

54. Evans, D. C., Schott, R. K., Larson, D. W., Brown, C. M. & Ryan, M. J. The oldest North American pachycephalosaurid and the hidden diversity of small-bodied ornithischian dinosaurs. *Nat. Commun.* **4**, 1–10 (2013).

55. McDonald, A. T., Espílez, E., Mampel, L., Kirkland, J. I. & Alcalá, L. An unusual new basal iguanodont (Dinosauria: Ornithopoda) from the Lower Cretaceous of Teruel, Spain. *Zootaxa* **76**, 61–76 (2012).

56. McDonald, A. T., Gates, T. A., Zanno, L. E. & Makovicky, P. J. Anatomy, taphonomy, and phylogenetic implications of a new specimen of *Eolambia caroljonesa* (Dinosauria: Ornithopoda) from the Cedar Mountain Formation, Utah, USA. *PLoS One* **12**, e0176896 (2017).

57. Prieto-Márquez, A., Wagner, J. R. & Lehman, T. An unusual 'shovel-billed' dinosaur with trophic specializations from the early Campanian of Trans-Pecos Texas, and the ancestral hadrosaurian crest. *J. Syst. Palaeontol.* **18**, 461–498 (2020).

58. Yang, Y., Wu, W., Dieudonné, P.-E. & Godefroit, P. A new basal ornithopod dinosaur from the Lower Cretaceous of China. *PeerJ* **8**, e9832 (2020).

59. Butler, R. J., Liyong, J., Jun, C. & Godefroit, P. The postcranial osteology and phylogenetic position of the small ornithischian dinosaur *Changchunsaurus parvus* from the Quantou Formation (Cretaceous: Aptian-Cenomanian) of Jilin Province, north-eastern China. *Palaeontology* **54**, 667–683 (2011).

60. Han, F.-L., Forster, C. A., Xu. X. & Clark, J. M. Postcranial anatomy of *Yinlong downsi* (Dinosauria: Ceratopsia) from the Upper Jurassic Shishugou Formation of China and the phylogeny of basal ornithischians. *Journal of Systematic Palaeontology* **16**, 1159–1187.

61. Boyd, C. A. The systematic relationships and biogeographic history of ornithischian dinosaurs. *PeerJ* **3**, e1523 (2015).

62. Madzia, D., Boyd, C. A. & Mazuch, M. A basal ornithopod dinosaur from the Cenomanian of the Czech Republic. *J. Syst. Palaeontol.* **16**, 967–979 (2018).

63. Herne, M. C., Nair, J. P., Evans, A. R. & Tait, A. M. New small-bodied ornithopods

(Dinosauria, Neornithischia) from the Early Cretaceous Wonthaggi Formation (Strzelecki Group) of the Australian-Antarctic rift system, with revision of *Qantassaurus intrepidus* Rich and Vickers-Rich, 1999. *J. Paleontol.* **93**, 543–584 (2019).

64. Barta, D. E. & Norell, M. A. The osteology of *Haya griva* (Dinosauria: Ornithischia) from the Late Cretaceous of Mongolia. *Bull. Am. Museum Nat. Hist.* **445**, 1–112 (2021).

65. Sues, H.-D., Evans, D. C., Galton, P. M. & Brown, C. M. Anatomy of the neornithischian dinosaur *Parksosaurus warreni* (Parks, 1926) from the Upper Cretaceous (lower Maastrichtian) Horseshoe Canyon Formation of Alberta, Canada. *Cretac. Res.* **141**, 105369 (2023).

66. Ronquist, F. & Huelsenbeck, J. P. MrBayes 3: Bayesian phylogenetic inference under mixed models. *Bioinformatics* **19**, 1572–1574 (2003).

67. Bapst, D. W. Paleotree: An R package for paleontological and phylogenetic analyses of evolution. *Methods Ecol. Evol.* **3**, 803–807 (2012).

68. R Development Core Team. R: A language and environment for statistical computing. (2013).

69. Lloyd, G. T., Bapst, D. W., Friedman, M. & Davis, K. E. Probabilistic divergence time estimation without branch lengths: Dating the origins of dinosaurs, avian flight and crown birds. *Biol. Lett.* **12**, 20160609 (2016).

70. Matzke, N. J. & Wright, A. Inferring node dates from tip dates in fossil Canidae: The importance of tree priors. *Biol. Lett.* **12**, 20160328 (2016).
